# A Review of Waterjet Cutting Research towards microAWJ and the Definition of the Waterjet Digital Twin

**DOI:** 10.3390/ma17061328

**Published:** 2024-03-13

**Authors:** Massimiliano Annoni

**Affiliations:** Department of Mechanical Engineering, Politecnico di Milano, 20156 Milano, Italy; massimiliano.annoni@polimi.it

**Keywords:** waterjet, waterjet cutting, process modelling, sensors, measurements, monitoring, control, microAWJ, high precision, digital twin

## Abstract

This review paper aimed to draw the red line passing through almost 25 years of research on waterjet cutting carried out at WJ_Lab, the waterjet laboratory of the Department of Mechanical Engineering of Politecnico di Milano. The purpose was not to just historically analyse the obtained scientific results by themselves but to make them even more useful by introducing the concept of the waterjet digital twin passing through the accuracy improvements due to microAWJ. This strategy effectively creates synergy among the topics and gives the opportunity to researchers in this field to both have an example of how research in industrial manufacturing processes can be guided by scientific and industrial needs, at least from the author’s point of view, and to appreciate how it can be made useful for further improvements by introducing a powerful concept as the digital twin.

## 1. Introduction

Waterjet cutting is an amazing manufacturing process with a wide range of advanced applications in many industrial fields such as mechanical, aeronautical, biomedical, electronic, marble and stone, composite materials, glass, ceramics, etc.

This paper aimed to give an insight into the waterjet cutting process by reviewing almost 25 years of research in this field carried out at WJ_Lab, the waterjet laboratory of the Department of Mechanical Engineering of Politecnico di Milano.

This paper was voluntarily self-referenced as its purpose was not to make a review of the state of the art about waterjet cutting but, as said, to elaborate on the research studies carried out at WJ_Lab in retrospect, to highlight which parts of that research can be considered important to the field and that are able to stand the test of time and prove effective and useful nowadays too. The scientific studies carried out by other researchers and kept as a reference when developing the reported WJ_Lab’s studies are cited in this review to complete the information given to the reader.

Cited scientific papers are numbered in order of appearance in the text, but the reader can understand the evolution of the research lines at the WJ_Lab by considering the years of the various publications belonging to a certain research category.

The considered research categories are:

- Process modelling (Section 2)
- Sensors and Measurements (Section 3)
- Monitoring and Control (Section 4)
- Component’s performance (Section 5)
- MicroAWJ (Section 6)
- Applications (Section 7)

Names of categories are self-descriptive, but it is worth highlighting in this introduction how, in the opinion of the author, they cover most of the aspects of the research on a manufacturing process, at least as far as the mechanical point of view is concerned.

This paper would like to be useful to researchers in the manufacturing field by giving an example of how research has been guided through the relevant topics characterising waterjet cutting, especially high-precision and microAWJ, but it also aimed to make this experience useful for the future by synthesising it and by outlining a concept existing already for other manufacturing processes, which is the waterjet digital twin.

The WJ_Lab research converges on this concept and it gains an even stronger technological significance with it (Section 8).

## 2. Process Modelling

Modelling is one of the main purposes of a manufacturing process as models give the possibility to understand, monitor and control manufacturing processes in the lab but also in industrial applications.

In this review, some research studies are taken as a reference as they set the theoretical base of waterjet cutting:

- Ref. [1] deals with the most important effects of water pressure on the waterjet cutting capability and defines orifice coefficients and mixing efficiency;
- Ref. [2] is a book organising the waterjet cutting knowledge in an effective and practical way;
- Ref. [3] is a fundamental study about the kerf formation that explains the abrasive waterjet cutting capability through the particles’ impact angle against the cutting front by introducing the so-called “cutting wear zone” at the top of the kerf, where the impact angles are small, and a “deformation wear zone”, at the bottom of the kerf, where impact angles are big. Ductile materials such as metals are better cut in the former zone, while fragile materials are better cut in the latter;
- Ref. [4] is a synthesis of the Mohamed Hashish’s studies that covers most of the waterjet cutting topics and represents a good lecture for beginners and experts. In particular, jet velocity, coherence, suction capability and back pressure are well introduced in this paper;
- Ref. [5] deals with the jet kinetic power density, the physical quantity that is more related to the jet cutting capability;
- Ref. [6] makes an overview of empirical models for estimating the cutting depth as an index of the cutting capability. Then it introduces an empirical model and an experimental procedure that not only predicts the cutting depth value based on the process parameters, but it uses it to calculate the relative cutting depth at which the roughness can be estimated. A useful economical model is also introduced to allow a technical-economical optimisation.

Modelling efforts carried out at WJ_Lab have been mainly based on empirical experiments carried out on the industrial plants available at Politecnico di Milano. Care has always been dedicated to organising and executing repeatable experiments to produce scientific results that could be useful for the waterjet research and industrial communities.

Scientific works have always been targeted to real applications more than speculative discussions and have been directed to consider the main governing components of a waterjet cutting plant such as pumps, mixing chambers, orifices and focusing tubes.

This section draws the red line among research works carried out at WJ_Lab that can be strictly categorised as modelling studies. Other sections include studies that could have a modelling part, but are more meaningful for other reasons (categories are reported in Section 1).

**Figure 1:**
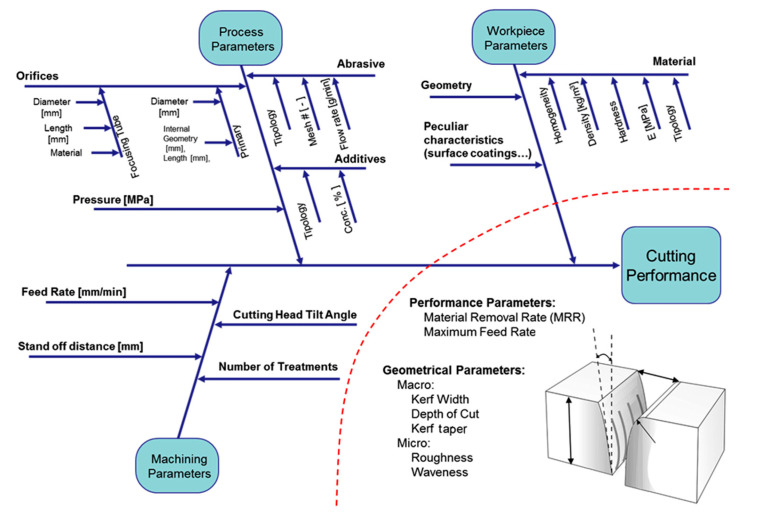
Waterjet Ishikawa diagram [7]. The waterjet cutting performance is described to the right of the red dotted line.

Figure 1 depicts the Ishikawa diagram that inspired many studies at WJ_Lab [7].

### 2.1. Kerf Taper Modelling

**Figure 2:**
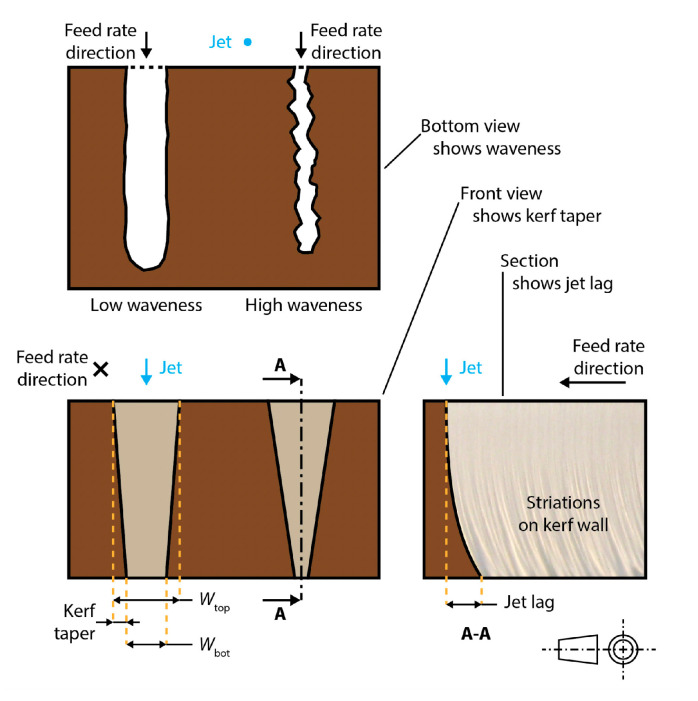
Representation of kerf waviness, kerf taper and jet lag [10].

The experimental work reported in [8] confirmed the dependence of the surface finish and kerf geometry (Figure 2) [9] on the typical AWJ parameters, and particularly on the feed rate. Its influence on the roughness and waviness evidences how, to get a high quality AWJ surface, low levels of feed rate are required, which is well known in the waterjet field. When the feed rate is low, the kerf taper reduces and could pass from a convergent shape (largest width at the top) to a divergent one (largest width at the bottom). The condition of null taper (corresponding to the “lower limit feed rate”) is particularly important for cutting near-net-shaped parts since kerf walls are parallel in this case. This study found a functional relationship between the AWJ process parameters and the lower limit feed rate on aluminium.

This research line went on to further develop the kerf taper model and couple it with other waterjet quality performance models [11]. The taper model is joined to the roughness prediction model proposed by other studies carried out at Politecnico di Milano [6]. Via this approach, it is possible to obtain the set of process parameters respecting the quality constraints under both the surface finish and the kerf geometrical shape points of view. The further step included in [11] was the integration of the taper model in the optimisation procedure of the proposed AWJ direct cost function allowing for the selection of the optimal set of process parameters under quality and economical constraints.

The analysis of the possibility to get the maximum kerf geometrical quality, identified with a null taper, led us to specifically consider the role of the abrasive mass flow rate.

The possibility to simultaneously control the abrasive mass flow rate and the feed rate in AWJ cutting has been studied in [12] making use of a device able to continuously control the abrasive mass flow rate during the cutting operations (see references [13,14,15] for the patents on abrasive feeding systems). When the feed rate must be reduced, for example when close to the changes of direction, some geometrical characteristics of the kerf may change, such as the kerf width and taper. In this case, it is possible to act on the abrasive mass flow rate, reducing it to compensate, under the taper point of view, the effect of the feed rate decreases. At the same time, the tolerance of the obtained shape can be fulfilled by means of a cutting path correction compensating the kerf width variation. Furthermore, the typical geometrical issues on the cutting path corners due to the jet deflection could be reduced by the continuous control of the abrasive mass flow rate. This approach is particularly useful when using 3-axis machines with no possibility of compensating the kerf taper by inclining the cutting head. The continuous control of the abrasive mass flow rate could widen the field of application of AWJ precision machining. 

### 2.2. Water Pressure Signal Modelling

Another fundamental waterjet parameter, the water pressure, has been studied under the point of view of the pressure signal generation, believing that a deeper knowledge on pressure fluctuation is needed to better understand the jet behaviour [16]. 

This study was the first of a series devoted to better understand the role of typical waterjet plant components on the cutting quality.

**Figure 3:**
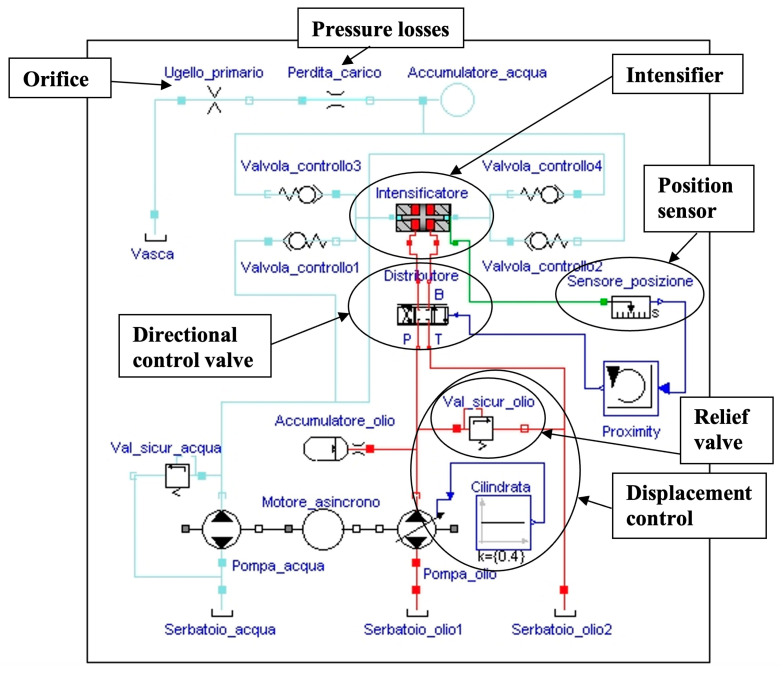
The double-acting intensifier pump (Flow Corporation 9XV-S, Kent, WA, USA) modelled with Dymola 5c (Modelica language) [16].

Pressure fluctuations are related to the pressure intensifier architecture, but also to the characteristics of the other components of the pumping system. A model able to simulate the pump behaviour and implemented with the Modelica language [17] was reported in [10] (Figure 3). The model was validated via an experimental campaign and is a powerful tool for pump designers and researchers.

### 2.3. Orifice Studies

**Figure 4:**
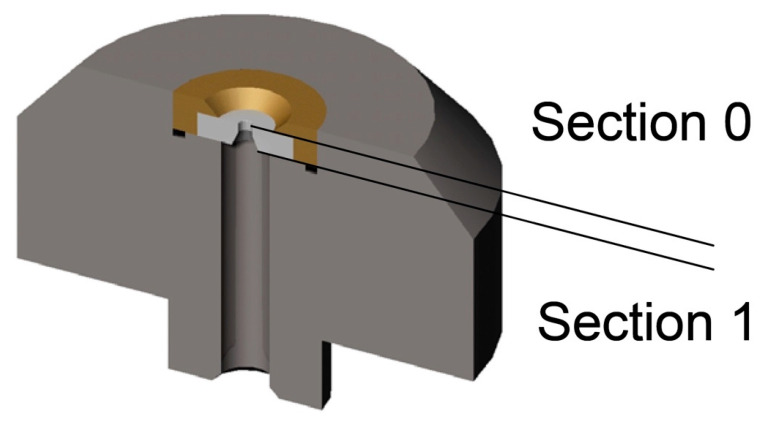
Typical waterjet orifice (more details in Section 5.1) [18].

After studying the “heart” of a waterjet plant, i.e., the pump, we focused on what we think of as the “brain”, i.e., the cutting head and, in particular, the orifice (Figure 4).

The performance of waterjet orifices depends not only on their nominal diameter but also on their internal geometry, which means inlet roundness, length of the cylindrical section (capillary section), length and angle of the cone section, ratio between inlet roundness and diameter, ratio between length of the cylindrical section and diameter and so on [19,20]. The study reported in [21] deals with the effect of diamond orifice geometry on the cutting performance and, in particular, with the effect of the position of the cone section. Two main configurations have been studied: cone-up orifices, which present the cone section at the entrance, and cone-down orifices, which present the cone section at the exit. The studied cone-down orifices can be considered as sharp-edged since they count on an inlet roundness close to zero, that is obtained thanks to the properties of diamond. Results reported in [21] pointed out how cone-up orifices, instead, count on a better coefficient of discharge but on a slightly lower velocity coefficient compared to standard sharp-edged orifices, which is consistent with the specific literature.

**Figure 5:**
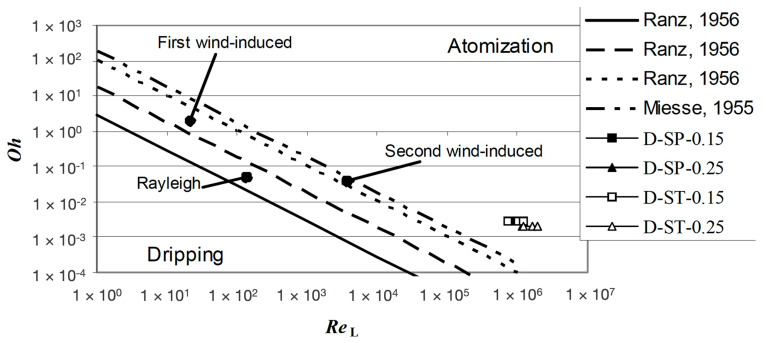
Ohnesorge diagram; tested conditions have been represented as D-SP-0.15, D-SP-0.25, D-ST-0.15, D-ST-0.25) (D: diamond; SP: special geometry; ST: standard geometry; 0.15: $d_{n}$ = 0.15 mm; 0.25: $d_{n}$ = 0.25 mm) [21].

This study is a good reference as it deals with the fluid-dynamics of nozzles through the main governing equations used in the field for calculating the waterjet velocity and flow rate (Table 1), the definition of nozzle coefficients (compressibility, velocity, contraction and discharge coefficients) [1] and the definition of relevant dimensionless numbers (Reynolds, Weber and Ohnesorge) that are useful for defining the jet break-up mechanisms (dripping, Rayleigh, first and second wind-induced and atomization, Figure 5) [22,23,24]. Finally, the hydraulic flip case is introduced, which is typical of waterjet sharp-edged orifices [25]. These orifices can create a stable and glass-like jet even at very high Reynolds numbers (10^6^–10^7^) thanks to the detachment of the water stream from the orifice walls due to both their sharp edge and a correct ratio (equal to 1) between length of the cylindrical section and diameter.

This study has been fundamental for WJ_Lab as it paved the way for following studies on microAWJ, where the stability and quality of the jet is of paramount importance (Section 6).

A laser Doppler velocimeter [26] was implemented to carry out the water jet velocity measurements reported in [21] (Section 3.2, Section 5.6).

The experimental procedure introduced in [21] has been further employed to experimentally highlight differences in the fluid-dynamic performances of orifices coming from different manufacturers in case of abrasive waterjet cutting of aluminium [27]. The coefficient of discharge alone is not sufficient to explain the cuffing performance of orifices, which indicates how other orifice features play a role. This study pointed out that it is necessary to study the jet behaviour in air (coherence), in addition to the orifice coefficients, to completely explain the orifice performance. This consideration is another step towards the understanding of the important factors enabling high-precision waterjet cutting (Section 6).

**Figure 6:**
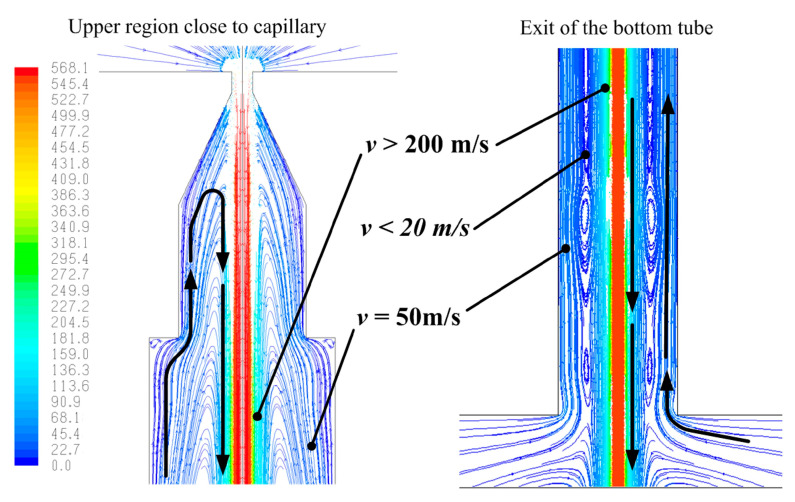
Path lines coloured by velocity magnitude [m/s]. The recirculation of the air inside the orifice exit tube is highlighted by black arrows [28].

**Figure 7:**
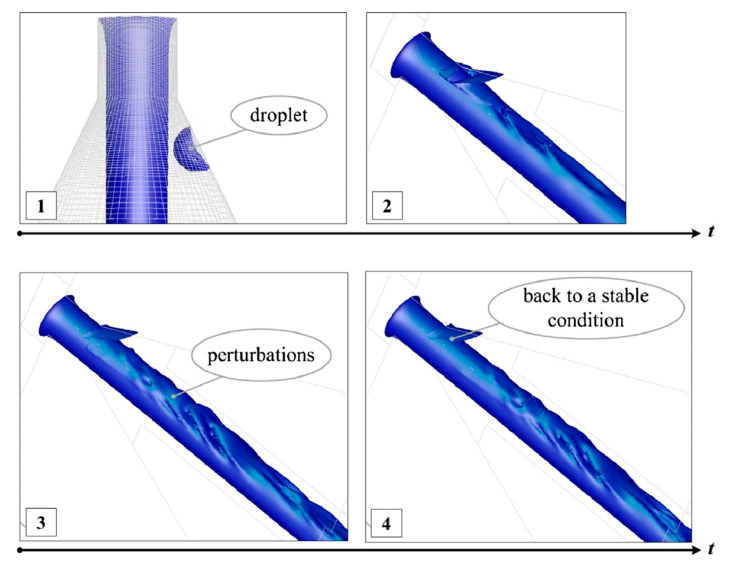
Inception of a jet instability caused by a droplet perturbation (flow time about 3 µs) [29]: (**1**) patching of a water droplet in the capillary region; (**2**) the droplet is initially deformed and dragged upwards by the air towards the capillary section until it gets in contact with the main jet; the “bridge” between the jet and the capillary wall suddenly creates a perturbation on the jet surface; (**3**) the perturbation increases; (**4**) the droplet is completely absorbed by the main jet and the “water bridge” disappears re-establishing the previous regime conditions.

**Figure 8:**
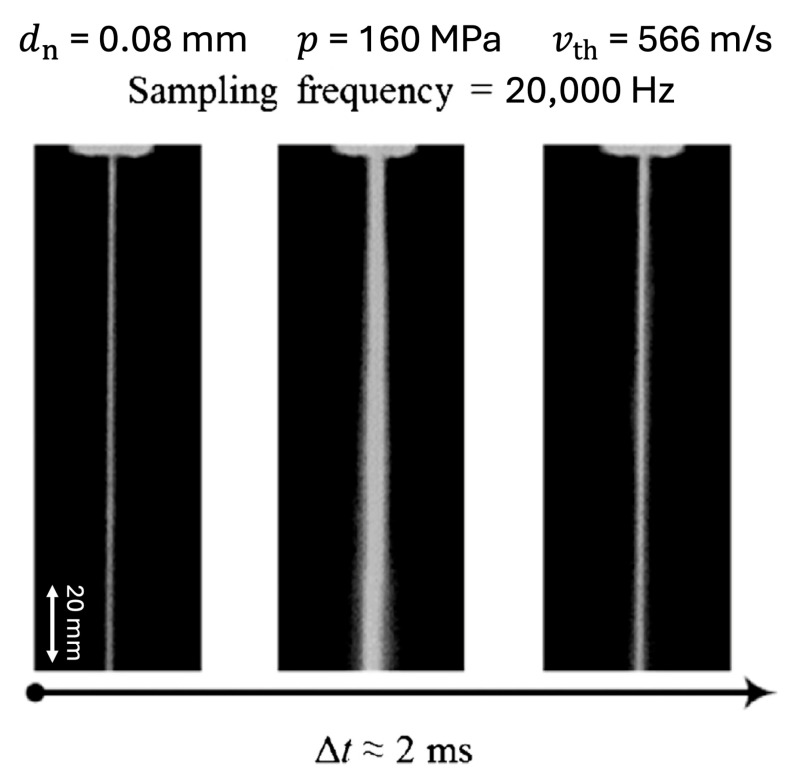
Instability of a pure waterjet recorded with a high-speed camera (HSC) [29].

It was clear at that moment that to better understand the jet behaviour, CFD simulation would have been a useful tool. Therefore, the research reported in [28] was carried out to apply numerical simulation to pure waterjet with the aim of investigating its creation and stability, achieving a deeper knowledge of the process and its disturbing factors, and improving our control capabilities. The internal geometry of the orifice plays an important role during the first instants of the jet formation affecting the jet break-up and the creation of droplets, which remain inside the orifice sticking or rebounding on the walls of the exit tube of the orifice. A CFD analysis was carried out to study the effect of the droplet collision with the main jet; jet break-up, early presence of water, condensed humidity or jet disturbances can create these water droplets, which then can be dragged upwards by the high-velocity air field created inside the orifice tube by the main water jet (Figure 6). Droplets can later collide with the main jet or be pushed up towards the capillary (the upper small orifice hole where the jet is created) causing local disturbances and loss of the hydraulic flip condition, which is crucial for the coherence of the jet (Figure 7). This random process effectively explains the instabilities that can be usually noticed by a naked-eye observation during and after the water jet formation (Figure 8). By studying this phenomenon, new concepts for an improved design of waterjet cutting head components have been gained on the way to high-precision applications. The simulation results were validated via means of a high-speed camera.

A subsequent study based on CFD simulation [29] went deeper into the analysis of disturbances and instabilities systematically affecting the jet structure, both during the jet formation and the cutting process.

**Figure 9:**
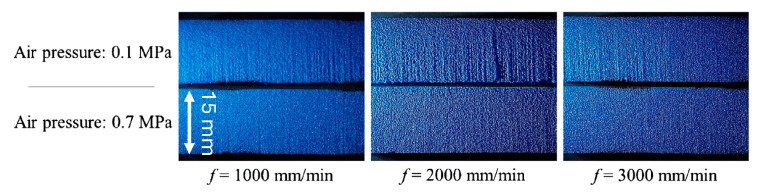
Air-assisted pure WJ cutting of Evazote^®^ (Zotefoams plc, Croydon, UK) with various combinations of air pressure and feed rate (*f*) (p = 200 MPa; $d_{n}$ = 0.08 mm).

These disturbances can be neglected in standard waterjet industrial applications, but they can play a relevant role in case of high-precision waterjet machining. The aim of the research presented in [29] was to develop an innovative system able to modify the orifice flow field by means of a simple modification of the standard cutting head geometry. The system allowed for the controlled injection of air inside the primary orifice through a lateral inlet port to prevent the jet instabilities and to adapt the level of jet coherence to the specific machining operation (Figure 9). The fluid-dynamic aspects of the outflow process [30] were investigated via a 3D numerical simulation [31] with the Ansys Fluent^®^ CFD solver. Since the geometrical domain and the perturbation dynamics are nonsymmetrical, a 3D model is necessary to correctly represent the fluid flow. The simulation domain included the orifice internal volume and a small volume at the entrance and at the exit of the orifice mounting (Section 5.1), whose CAD models were defined starting from accurate measurements. According to the Fluent 12.0 Theory Guide [32], the best way to simulate the mechanism of a water jet discharging in air, for our purposes, is to set up a time-dependent multiphase turbulent flow model with the addition of surface tension. Volume of fluid (VOF) is used as a multiphase model since it is particularly suitable for tracking the interface between two nonmiscible fluid phases (water and air), which are considered as incompressible in the present simulation. Surface tension is added as well for a correct evaluation of all the forces acting on the jet surface. The rest of the settings have been selected to obtain the best results in terms of simulation convergence and accuracy, as suggested in the Fluent 12.0 User’s Guide [33]. A comprehensive model settings summary is reported in Table 2.

$p_{\mathrm{up}}$ in Table 2 is the upstream water pressure, $p_{\mathrm{dowm}}$ is the downstream pressure and $p_{\mathrm{air}}$ is the air pressure at the lateral inlet port [29]. In addition, since the resolution of the VOF surface tracking is strongly dependent on the mesh resolution, a mesh refinement was performed in the interested areas (i.e., the orifice capillary region and the jet surrounding) to find the best compromise between results accuracy and computational time.

Considerable experimental efforts were provided to validate the numerical model and finally evaluate the system performance on real case studies. Figure 9 reports cutting results on Evazote^®^ (a closed-cell cross-linked ethylene copolymer foam; average density: 50 kg/m^3^). Air was injected through the orifice lateral inlet port at two different pressures: 0.1 MPa creates a coherent jet, while $p_{\mathrm{air}}$ = 0.7 MPa produces a divergent jet structure resulting in a counterintuitive kerf quality improvement [29]. These considerations have been useful for the next improvements towards microAWJ (Section 6).

Waterjet cutting has always been a promising technology because of its extreme flexibility, even if it often suffers a lack of control on its process parameters, especially if compared to technologies such as laser cutting or electrical discharge machining. WJ_Lab invested in these studies on jet formation and stability to improve the jet capability control and come to an improved precision performance.

At the end of 2014, the WJ_Lab studies on the jet formation and the awareness about the plant component selection in view of an improved waterjet cutting accuracy led to the idea to found a spin-off company, WatAJet s.r.l. (www.watajet.com, accessed on 28 February 2024), working in the field of high-precision waterjet cutting (see also Section 6).

More recently [34], some research efforts have been devoted to test a theoretical model for calculating abrasive waterjet (AWJ) cutting parameters to reduce the shape deformation in curved cutting trajectories. The model is based on previous studies about the depth of cut [35] and the cutting front shape produced by the jet when cutting thick parts [36,37]. A method aimed at reducing the shape distortion caused by AWJ in curved geometric features was developed and tested. It was unambiguously demonstrated that the jet markedly reduced the shape deformation in curved geometric features when it was tilted according to the proposed method. Such an error compensation procedure can be already applied to commercial machines.

## 3. Sensors and Measurements

Waterjet system sensorisation provides opportunities in terms of system characterisation, process monitoring and control on the way to an improved final cutting quality. The acquisition and analysis of signals such as oil and water pressures, water flow rate, intensifier piston velocity, abrasive mass flow rate, air pressure and flow rate in the mixing chamber and so on, are useful for understanding the behaviour of the system and for detecting malfunctioning situations (Table 3).

This section involves the research studies devoted to the definition of the sensor and measurement equipment and is strictly related to the monitoring section (Section 4) that is dedicated to the development of monitoring strategies. 

### 3.1. Pump Electric Power Signal

In [38], the correlation between the electric input, the mechanical and the fluid-dynamic signals [39,40] were analysed with the aim to design an online and nonintrusive monitoring and diagnostic system.

**Figure 10:**
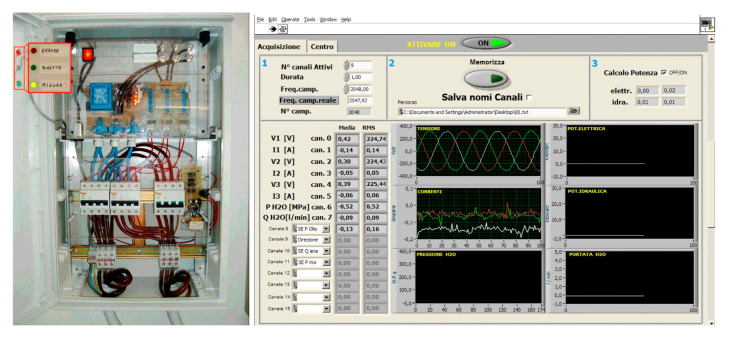
(**Left**) three-phase power transducer (National Instruments PCI 6034E, input: ±10 V, Sampling frequency: 200 kHz). Current: LEM; LA−100 P (100 A). Tension: analogue device; AD215BY. Sensors implemented in a self-produced three-phase power transducer board [18]. (**Right**) acquisition panel (LabView 6.1).

**Figure 11:**
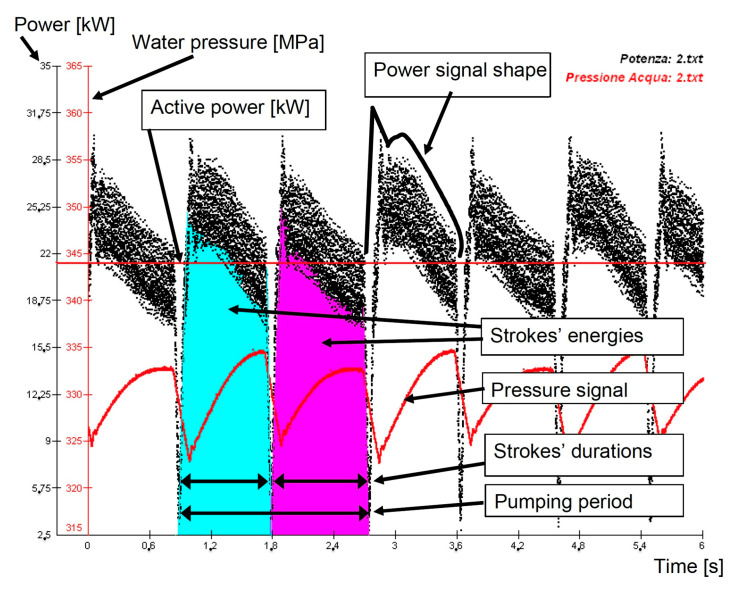
Reference signal (A-0.30 330) ($d_{n}$ = 0.30 mm; p = 330.2 MPa; P = 21.57 kW; f = 0.54 Hz; $Q_{w}$ = 2.37 L/min; $c_{d}$ = 0.69; *K*_e_ = 98.94%; *K*_p_ = 94.74%; *η* = 0.60). This condition has been useful for carrying out the comparisons of different operating conditions [18].

The studied waterjet plant was equipped with sensors [41,42] to acquire the signals of the most important parameters describing its fluid-dynamic behaviour: oil pressure, water pressure, water volume flow rate and piston velocity (Table 3). To perform the combined analysis, line voltages and currents were transduced and acquired in a measurement section located at the motor input (Figure 10). It was demonstrated that some synthetic indexes (*K*_e_: the ratio between the minimum stroke energy and the maximum stroke energy in a pumping period; *K*_p_: the ratio between the two different strokes’ durations; see also Section 4.2) can be calculated from the electrical motor power signal to describe the waterjet plant behaviour without acquiring other fluid-dynamic signals. For example, the water mass (and volume) flow rate can be estimated, under some hypotheses, by means of the pumping frequency calculated on the power signal (Figure 11).

### 3.2. Laser Doppler Velocimetry

**Figure 12:**
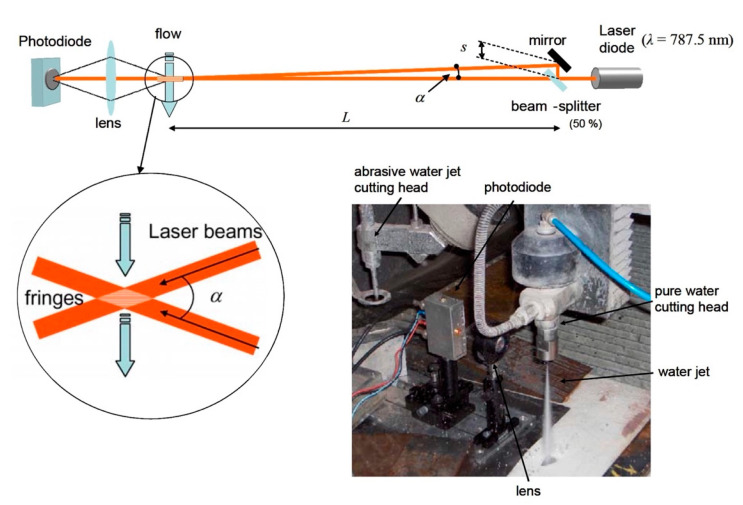
Sketch of the implemented laser Doppler dual-incident-beam velocimeter in reference-beam configuration [44].

An important instrument designed and developed at WJ_Lab for acquiring information on waterjet velocity was the laser Doppler velocimeter described in [43,44] (Figure 12).

The laser Doppler principle [26] is particularly suitable, in respect to other techniques [45,46,47], for velocity measurements of fluids where a noncontact approach is needed to not modify the flow state. In case of application to high-velocity water jets, the characteristics of this technique make it the most promising experimental procedure for accurate and nonintrusive velocity measurements, useful for gaining a better understanding of the fluid-dynamic phenomena, even in the presence of an abrasive [48,49].

The LDV technique was implemented to evaluate waterjet velocity in waterjet and abrasive waterjet cutting plants. The knowledge of waterjet velocity is fundamental to determine the system efficiency. LDV allows users to experimentally measure the waterjet velocity and therefore calculate the orifice coefficients described in Section 2.3.

When small particles suspended in the fluid or ripples on the jet surface pass through the fringe pattern created by two incident laser beams, they provide a diffused light exhibiting an amplitude modulation at a frequency *f*, simply depending on the speed v of the particles and fringe spacing *δ*: *f* = v/*δ*.

**Figure 13:**
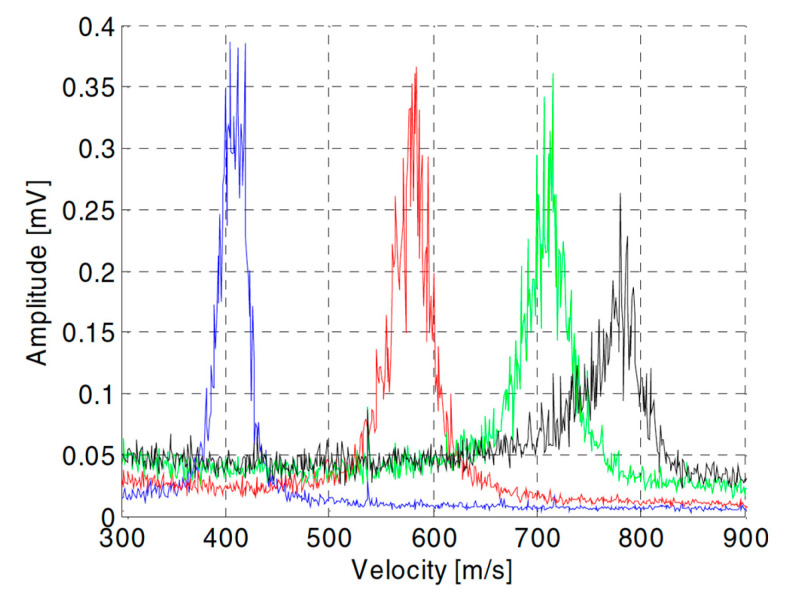
Signal acquired for pressures 100 MPa (blue), 200 MPa (red), 300 MPa (green) and 350 MPa (black) for a 0.15 mm standard orifice [43].

Figure 12 reports the developed LDV setup and Figure 13 shows the signals acquired for a 0.15 mm orifice at different water pressures.

The setup procedure for the laser Doppler velocimetry equipment requires competence regarding the error sources and care in the preliminary adjustment, as well as of the necessary conditions to evaluate the measurement uncertainty. A repeatable, operator-independent and quantitative setup procedure has been developed in [50] in the case of a laser Doppler dual-incident-beam velocimeter in reference-beam configuration on the way to a complete characterisation of the measurement instrument. Some reference literature on waterjet velocity measurements [51] and laser Doppler techniques [52] was studied on purpose. Determining the measurement uncertainty of a self-developed instruments is a necessary step for its use in scientific studies. 

## 4. Monitoring and Control

Monitoring and control are two fundamental activities when trying to improve every manufacturing process. This section discusses the WJ_Lab’s studies devoted to acquiring information on the process during machining and the attempts to keep the relevant parameters under control.

### 4.1. Abrasive Mass Flow Rate and Feed Rate 

One of the first process parameters considered to improve the manufactured parts through monitoring and control is the abrasive mass flow rate [2,53].

The importance of abrasive mass flow rate has been pointed out in [54,55] also including the effect of its fluctuation on the microgeometrical quality of the obtained surfaces. Abrasive mass flow rate closed-loop control systems are a valid solution for achieving high-quality cutting results, especially if the feed rate is simultaneously and opportunely controlled at the path corners [56]. 

In fact, both the feed rate and the abrasive mass flow rate show their remarkable effect on the kerf width and taper, so it is clear that if one of these parameters is constrained, as it happens when the feed rate must be reduced by the numeric control close to a corner, the other parameter could compensate to keep the required kerf characteristics. This opportunity was evaluated via an experimental campaign focused on the combined effects of the abrasive mass flow rate and the feed rate in particular cutting situations [56]. 

**Figure 14:**
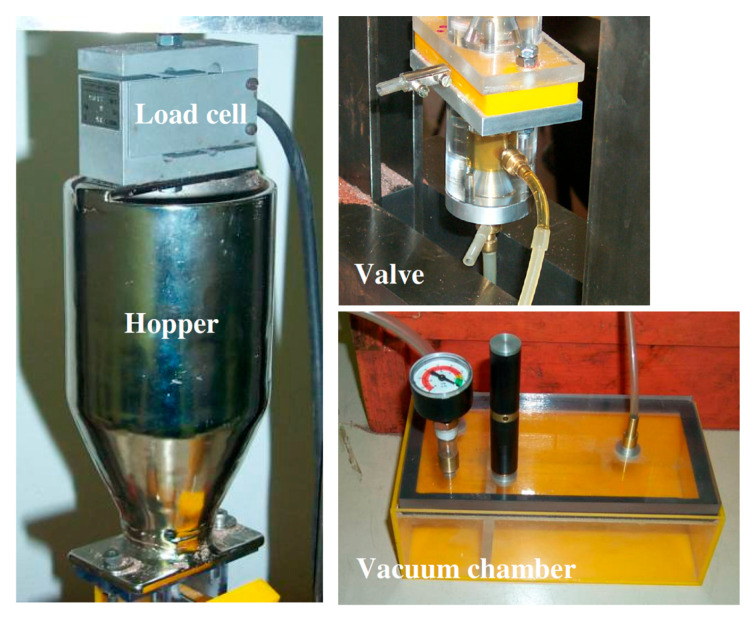
Main components of the abrasive mass flow rate closed-loop continuous control system [12].

The study reported in [12] describes the abrasive mass flow rate closed-loop control system that was developed and patented for this purpose [13] (Figure 14).

### 4.2. Pump Electric Power Signal

A series of papers have focused on the definition of synthetic indexes based on the pump’s instantaneous electrical power and its behaviour in time [57] (Section 3.1). In fact, it was demonstrated how the electric power signal is correlated to mechanical and fluid-dynamic signals. A set of indexes were calculated starting from the power signal and a “footprint” of a waterjet system in normal working conditions was obtained [58,59] (Figure 11):

- Active power: this parameter depends on the assigned working condition, once the orifice diameter and the water pressure values are assigned;
- *K*_e_: it is the ratio between the minimum stroke energy and the maximum stroke energy in a pumping period. It takes into account the asymmetry of the power and water pressure signals;
- *K*_p_: it is the ratio between the two different strokes’ durations;
- Pumping frequency (*f*) (evaluated from the instantaneous power): it is directly related to the water mass flow rate in the water circuit.

An online and nonintrusive monitoring and diagnostic system for this kind of device has been designed by implementing an algorithm based on the proposed “footprints” [60].

The same methodology can also be applied to the characterisation and comparison of the WJ plants’ components. In fact, [59] tested it on the characterisation and comparison of waterjet orifices as one of the most important WJ components.

Moreover, a procedure based on the analysis and classification of the instantaneous power signal features is presented in [61], while [62,63,64] show how the electrical power signal includes all the necessary information to characterise the system working condition and predict incoming faulty behaviours [65,66,67]. 

**Figure 15:**
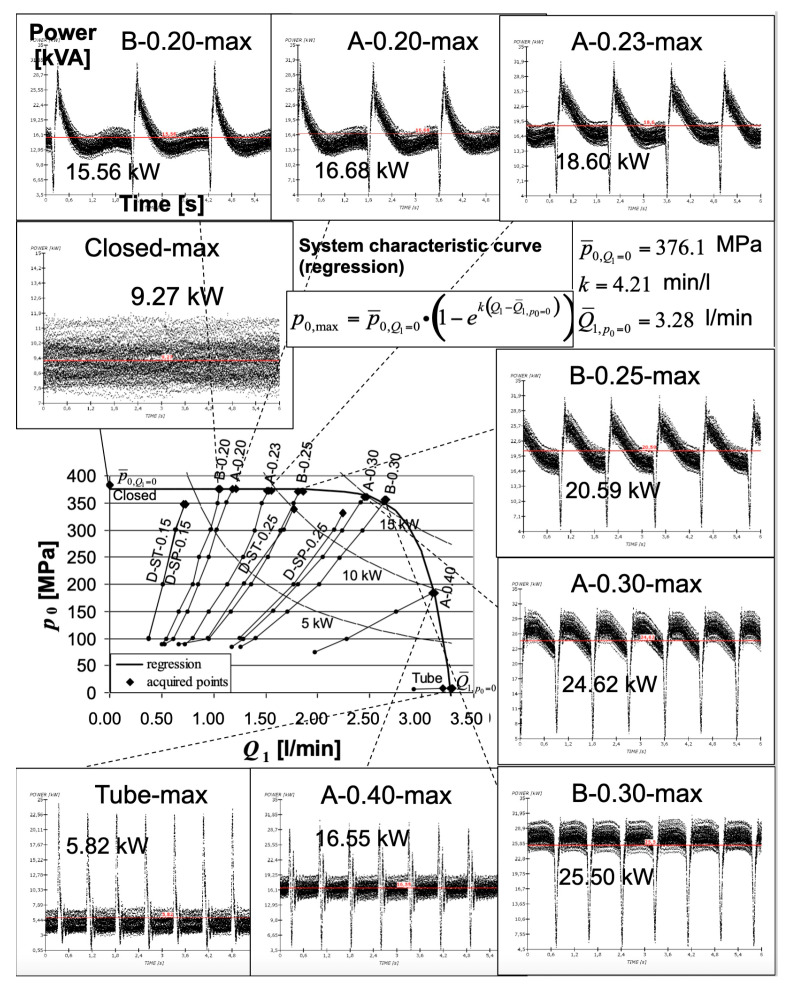
Power signals for different orifices at maximum pressure; orifices’ characteristic curves; system characteristic curve (A, B = orifice types; D = diamond orifice; ST = standard internal geometry; SP = special internal geometry; 0.15, 0.20, 0.23, 0.25, 0.30, 0.40 = tested orifice diameters (mm); max = maximum water pressure) [18].

Figure 15 highlights how the pump’s instantaneous electrical power signal characterises the various working conditions of a cutting plant [18,58,60]. The same picture is also important since it reports the typical characteristic curve of a waterjet intensifier pump that was obtained as the envelope of the working conditions of various orifices with different diameters at the maximum water pressure given by the pump. It is possible to notice how the “B-0.30” orifice can release the maximum power by working at the knee of the curve. This is the suggested working condition for a “separation cut.” The pump characteristic curve is fundamental when dimensioning a waterjet cutting operation, especially when multiple orifices are used in parallel, but unfortunately it is not always provided by pump manufacturers.

### 4.3. Pump Plungers’ Displacement

When waterjet cutting end users are interviewed, most of them point out that the most critical problem of waterjet machines is the reliability of the system components, together with the difficulty in estimating their lifetime. As far as the UHP (ultra-high pressure) intensifier is concerned, there are several components that work under extreme fatigue conditions, considering that the pressure inside the cylinders can reach 400 or even 600 MPa. Different failure scenarios can be envisaged, leading to different pattern deviations from nominal behaviour conditions. 

In [68], a correlation analysis on multiple signal features with the health status of the machine is presented [69,70]. Then a multisensor-based monitoring approach is discussed and tested on a single-acting multiple plunger pump. This approach was based on the usage of control charts for in-control region definition and possible detection of faults.

An interesting result was that it was possible to distinguish among three faulty components when the cylinder was in the out-of-control state: cylinder body, outlet valve body and outlet valve housing.

A new approach was investigated in [71] for the online health condition assessment of both UHP pump and cutting head components by using a single type of information source, i.e., the plunger displacement signal. Some relevant literature was considered for AWJ monitoring systems [72,73,74,75], statistical analysis [76,77], condition monitoring [78] and predictive maintenance [79]. A multivariate analysis of variance (MANOVA) [69] was performed to study the effects of actual faulty components on the acquired signals during AWJ cutting. The results demonstrated that the plunger displacement signal is suitable for detecting and identifying critical faults in WJ/AWJ cutting systems.

### 4.4. Monitoring the Waterjet Cutting Performance at the Focusing Tube

Recent studies at the WJ_Lab have been devoted to investigating the possibility of monitoring the waterjet cutting plant by sensorising the focusing tube [80]. This is an interesting possibility as it is noninvasive and allows for the acquisition of information at the very end of the abrasive waterjet formation. Many research papers are present in the literature on AWJ condition monitoring [72,81], cutting depth monitoring [82], surface quality prediction through acoustic sound pressure monitoring [83], vibration monitoring [84] and nozzle wear [85,86].

The experimental evidence reported in [87], based on patents reported in [88] and [89], proves that the operational vibration monitored by means of two accelerometers installed at the tip of the focusing tube is well related to the kinetic power of the abrasive particles $P_{\mathrm{part}}$, i.e., the only portion of the jet power that is responsible for the material removal in abrasive waterjet cutting (Table 4) [5]. 

**Figure 16:**
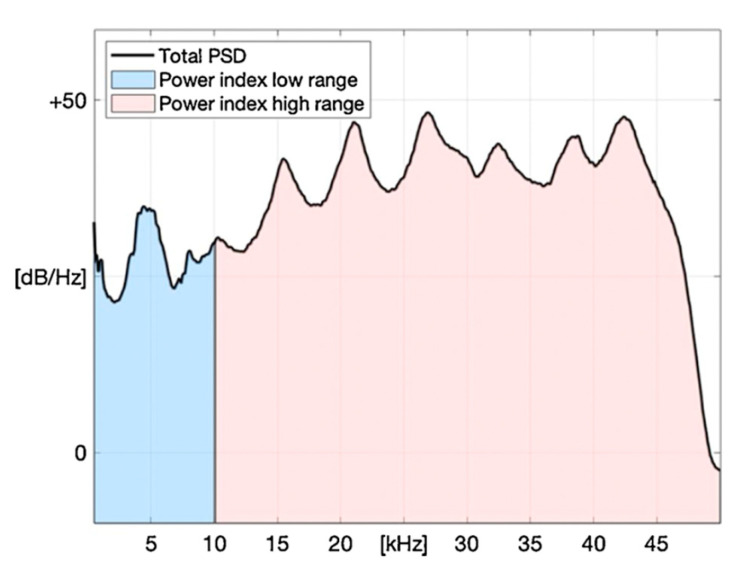
Total PSD and power indices (p = 330 MPa, ${\dot{m}}_{a}$ = 300 g/min). dB reference: 1 × 10^−6^ m/s^2^ [87].

The following method is proposed for extracting an indicator of $P_{\mathrm{part}}$ from the operational vibration delivered by the instrumented focusing tube, which is hereafter referred to as the power index:

1. Once the AWJ cutting machine has been started, the operational vibration is monitored by means of the two accelerometers at the focusing tube;
2. Transient vibration phenomena occur within the first 5 s after the cutting process start-up. The transient period is truncated from each time signal, leaving only the steady portion of 10 s for the next processing steps;
3. For each signal, the corresponding power spectral density (PSD) is computed;
4. The two PSDs are summed into a total PSD (Figure 16). Finally, the integral of the total PSD is computed in two predetermined frequency intervals (low range and high range). This integral constitutes the power index. Low range corresponds to the operational range of the accelerometers, apart from the 10–500 Hz range that is not important for the investigated phenomena. The high range reaches up to the Nyquist frequency of the acquisition module where the accelerometers operate out of their specifications, hence a signal distortion should be expected. Moreover, it is expected that water contributes more to the low frequency range, while abrasive particles provide their vibration signature above 10 kHz.

This approach was validated by means of an experimental investigation, where the abrasive waterjet was fired at different water pressures and abrasive mass flow rates, providing different kinetic powers.

**Figure 17:**
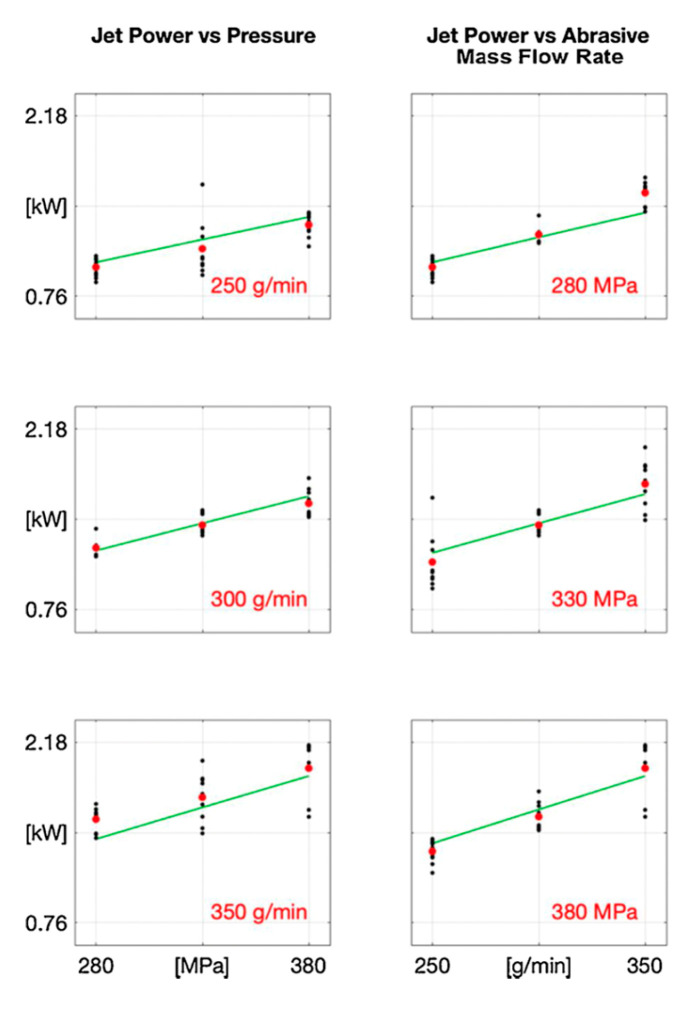
Comparison between theoretical jet power and power index values (high range). Green lines: theoretical trends. Black dots: power indices. Red dots: arithmetic averages [87].

In the high range, the power indices increase with both abrasive mass flow rate and water pressure, providing a robust and accurate indicator of the theoretical jet power (Figure 17). In the low range, the power indices do not fit well the theoretical trend. 

The information delivered enriches the process knowledge, thus paving the way for significant improvements ranging from closed-loop control strategies for the water and abrasive feeding systems to actions that support operators in compensating drifts of the jet cutting capability. The expected impact is an improvement of process automation and stability, as well as an enhanced process traceability.

### 4.5. Inline Focusing Tube Wear Progression Monitoring

**Figure 18:**
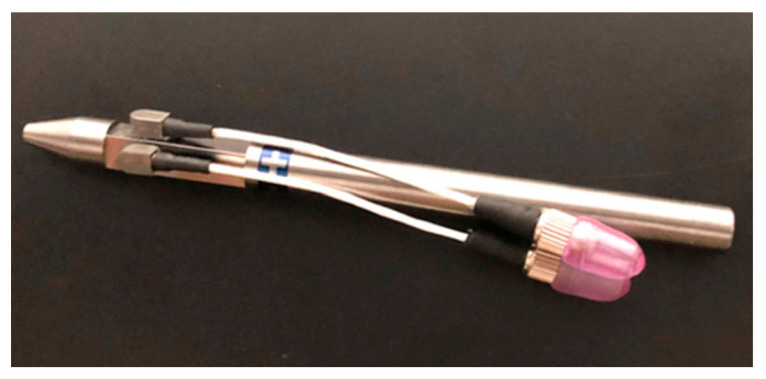
Focusing tube with special geometry and onboard accelerometers [96].

The waterjet focusing tube is a critical component, as its fast wear progression requires constant human supervision for promptly detecting detrimental effects on the cutting performance [90]. Reference papers can be found in the literature on AWJ process monitoring [91], focusing on tube erosion [92,93,94] and operating costs [95]. The research reported in [96] described an innovative approach based on the focusing tube operational vibration for inline monitoring of the wear progression of a waterjet focusing tube by means of an accelerometer installed on its tip. This result was achieved by two separate studies. The first investigated the sensitivity of the focusing tube’s first mode frequency to the wear progression, while the second demonstrated the possibility of tracking this frequency from the inline vibration signal delivered by the accelerometer, during operation. The presented setup makes use of low-cost sensing hardware that can be easily retrofitted into the design of waterjet focusing tubes (Figure 18). The information delivered is expected to tackle end-user requirements for improved process automation.

### 4.6. Airborne Acoustic Emission Monitoring to Assess the Waterjet Cutting Capability

Abrasive waterjet cutting is a manufacturing technology making use of a high-speed waterjet with abrasive particles in suspension, for cutting materials with different mechanical properties. Product quality requirements are pushing towards an improvement of tracking and stabilisation methods of the relevant process variables. Amongst those, the jet kinetic power defines the cutting capability and has a significant impact on the final cut features. This variable is subject to relevant fluctuations versus time. However, the current state-of-the-art process does not provide means for its inline monitoring. 

A step ahead on the possibility to effectively monitor the jet cutting capability has been made with the study reported in [97], where the aim was to monitor the airborne acoustic emission [98] of an abrasive waterjet cutting head [99] and to investigate its correlation with the jet kinetic power [5]. The investigation was carried out by means of factorial studies, in which the jet was fired at various water pressures and abrasive feed rates, providing different kinetic powers. The acoustic emission was synchronously monitored by means of a condenser microphone installed on the cutting head. Data at frequencies above 40 kHz were found to constitute a robust and selective acoustic signature of the airborne jet. The acoustic signature has proven to be an effective inline indicator of the jet kinetic power and its pressure-induced variations, whilst abrasive-induced variations remain undetected. A calibration procedure was presented, for translating the acoustic data into the jet kinetic power. Overall, the presented method constitutes a robust tool for monitoring pressure-induced variations of the jet cutting capability.

### 4.7. Waterjet Force Signal Monitoring

The forces produced on the workpiece during abrasive waterjet machining can yield some valuable information. Reference literature can be found on the erosion of metals [100], AWJ cutting of ductile materials [101], jet lag [102] and water-air-abrasive energy transfer [103]. Previous studies on waterjet force signals pointed out how the tangential-to-normal force ratio (TNR) is an appropriate quantity for monitoring purposes [104]. 

A special waterjet force measuring device designed and produced in the past was used for the research presented in [105] to validate the use of TNR. 

During cutting operations, the jet exerts a force on the workpiece consisting of three components. The tangential force (TF) acts parallel to the direction of the feed rate. It is assumed to correspond to the cutting force (CF). The lateral force (LF) acts perpendicular to TF in the same plane. The normal force (NF), perpendicular to TF and LF, includes the deformation force (DF). The lateral force is not an important factor in the case of linear cuts, as its value is usually 50–100 times lower than that of the TF. The study reported in [105] summarises the most important findings acquired on AWJ cutting machines with tilting cutting heads. TF and NF can be respectively linked with the cutting and deformation wear of the material inside the kerf. Therefore, the corresponding forces were measured and their ratio (TNR) were compared for several metallic materials considering different declination angles and jet axis tilting angles to verify the hypotheses predicted from the theory: (i) TNR strongly depends on the actual-to-limit traverse speed ratio; (ii) TNR relates to the cutting-to-deformation wear ratio inside the kerf; (iii) TNR value changes when the jet axis tilts towards the traverse speed direction. The results are sufficiently consistent to allow expectations that force measurements can be used to online monitor the AWJ machining process and even online control.

The findings of this research support the previously predicted and published assumptions that the cutting head tilting enables an increase in the cutting wear mode inside the forming kerf, making the process more efficient.

## 5. Component’s Performance

This section includes the research works on the characterisation of the waterjet plant components’ performance carried out at WJ_Lab.

This activity involves instruments, methods and knowledge developed in the other research categories, so proper links will be made to other sections of this paper.

### 5.1. Orifice Performance

As already pointed out in Section 2, the orifice is one of the most relevant components of a waterjet cutting system. For this reason, a good part of the WJ_Lab’s research has been devoted to modelling and characterising its role.

In this section, the main testing and characterisation studies are reported and commented on.

The set of relevant equations (Section 2.3, Table 1) and a first description of the experimental equipment and the experimental procedure used for characterising the orifice performance in terms of effectiveness and efficiency are reported in [106]. Other relevant literature can be found in [107,108,109].

It was clear how waterjet velocity measurements were necessary to complete the orifice characterising procedure so a novel laser Doppler velocimetry (LDV) technique was designed and implemented (Section 3.2). 

Various studies were dedicated to setting up a suitable LDV procedure for experimentally obtaining the orifice coefficients [110,111,112,113] whilst also considering the interesting case of a broken orifice [114] that produces a better value of coefficient of discharge, but a lower value of velocity coefficient in respect to an orifice in optimal working conditions, and for classifying the orifice working status [115] as regular or faulty.

**Figure 19:**
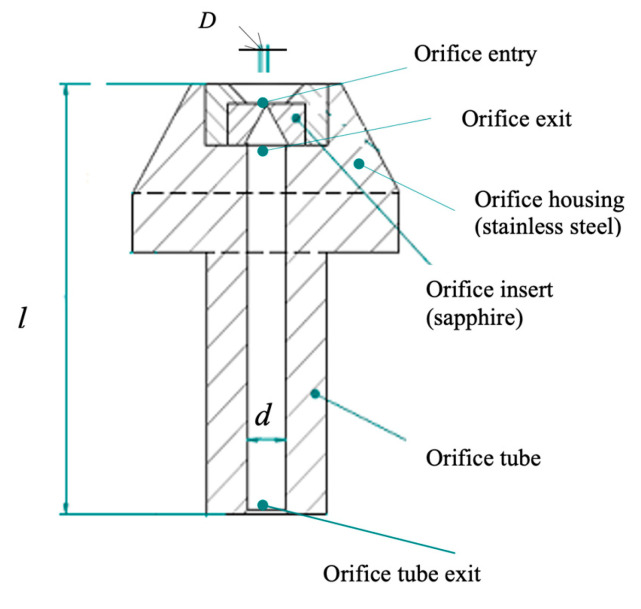
Section of an orifice and orifice housing assembly [116,123].

**Figure 20:**
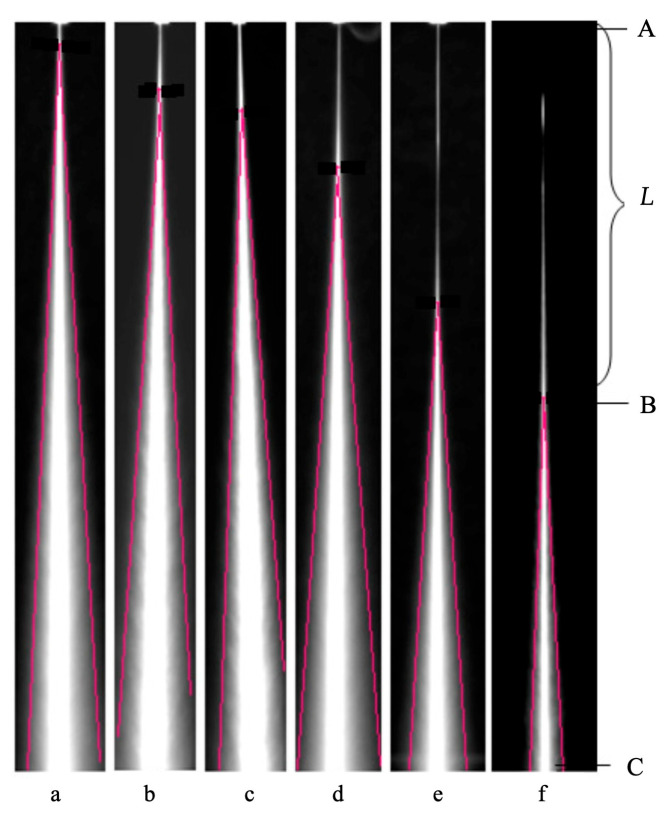
Effect of orifice housing length and diameter on the jet stability (experimental). AB: Compact zone; BC: disintegration zone; *L*: compact length; *p* = 220 MPa; *D* = *d*_n_ = 0.08 mm and (**a**) *d* = 0.7, *l* = 12.5 mm, *L* = 3.3, (**b**) *d* = 1.3, *l* = 12.5 mm, *L* = 6.7, (**c**) *d* = 1.3, *l* = 8.5 mm, *L* = 10.0, (**d**) *d* = 0.7, *l* = 8.5 mm, *L* = 16.7, (**e**) *d* = 1, *l* = 10.5 mm, *L* = 33.3 and (**f**) *d* = 0.5, *l* = 6 mm, *L* = 46.37 [116].

The effect of waterjet orifice housing geometry downstream of the orifice (Figure 19 and Figure 20) on the stability of a pure waterjet is presented in [116] with a view to enhancing the performance of contour cutting of foam materials in the seals and gaskets industry. Some relevant papers on the jet stability [117,118], multiphase jets [119], CFD analysis [120] and water jet measurements [121] were taken as a reference. CFD analysis was performed and it was found that the velocity magnitude and coherence of the jet depend on the geometry of orifice housing, in good agreement with experiments (Figure 20). 

The overexposed steady state images of the jets were obtained for different orifice housing tube diameters and lengths at the same conditions using a SensiCam (Excelitas Technologies Corp., Waltham, MA, USA) high-speed CCD camera. A flash duration of less than 1 μs was used to provide illumination for capturing the images of the moving jets.

Figure 20a–f show the typical disintegration pattern of a jet at the water pressure *p* = 220 MPa and orifice diameter *D* = *d*_n_ = 0.08 mm, when the orifice housing diameter and length are varied according to the caption of Figure 20 and the definitions of Figure 19.

Based on the imaging experiments, it was found that a jet typically consists of two zones (Figure 20f), a compact zone AB (corresponding to a compact length *L*), where the jet shows good coherence, and a disintegration zone BC where the jet evolves into a disintegrated jet. It should be noted that the transition from one zone to the other is gradual. The straight lines of Figure 20 were drawn along the periphery of the divergent jet profile. The point where they meet was conventionally taken as the transition point between the compact and the disintegration zones (point B).

The compact lengths *L* changed by varying the orifice housing geometry. Figure 20 reports the studied cases from the smallest value of *L* to the biggest. According to [122], the disintegration of a bulk liquid into small drops is caused by the loss of stability when waves develop on the liquid surface.

When a liquid jet emerges from an orifice as a continuous body, a competition takes place on the jet surface between the cohesive and disruptive forces, giving rise to oscillations and perturbations. The air friction first acts inside the orifice tube and, subsequently, downstream of its exit. The magnitude of this interaction depends on the tube diameter and length. Surface tension is active as a cohesion force that restrains the liquid from breaking up into drops. In contrast, aerodynamic forces acting on the liquid surface promote disruption. Break-up occurs when the magnitude of the disruptive forces exceeds the surface tension. The role of water viscosity, on the other hand, is to inhibit the growth of instabilities delaying the onset of disintegration [117].

Subsequent experimental work was carried out to show how the geometry of the orifice housing also plays an important role in the accurate contour cutting of foam material. A combination of high water pressure, small orifice exit tube diameter *d* and high orifice housing length *l* generates a smaller kerf taper at equal stand-off distance.

These considerations have been important for subsequent industrial applications of foam gasket cutting as they allowed for the proper selection of the orifice by also considering the housing geometry (e.g., length of the orifice exit tube).

### 5.2. Mixing Chamber Performance

Cutting heads are core components of abrasive waterjet cutting plants, which dramatically affect the achievable cutting performance. Therefore, investigating their characteristics is fundamental to achieving an efficient design and improving the AWJ technology. In [123,124,125], computational fluid-dynamics models for ultrahigh velocity waterjets and abrasive waterjets were established using the ANSYS Fluent^®^ software (version 6.3.12 used in [123,124]). Jet flow dynamic characteristics inside a cutting head were simulated under unsteady state, turbulent, two-phase and three-phase flow conditions. Water and particle velocities were obtained under different operating conditions to provide an insight into the jet characteristics and to study the effect of the cutting head geometry. The comparison with experimental data showed the accuracy of the numerical simulations in predicting the cutting head performance, as well as revealing the effect of operating conditions. Moreover, the fact that results obtained with two different geometrical models (2D-axisymmetric and 3D) were similar reveals that the flow pattern does not depend much on the position of the abrasive and air suction zone. This investigation improves the understanding of the flow inside the AWJ cutting head and provides information for designing this component to achieve optimum performances.

This study, together with [29], have been fundamental for the WJ_Lab to achieve awareness about the role of the geometry of the cutting head components (orifice, mixing chamber and focusing tube) on the jet stability in space (coherence) and in time. This knowledge paved the way towards the microAWJ developments (Section 6).

### 5.3. Polymeric Additives to Improve the Pure Waterjet Performance

A typical topic in the waterjet cutting field is the role of polymeric additives on the cutting performance [122,126,127]. WJ_Lab is a research lab, but with a particular attention to the industrial needs, so some efforts were dedicated to study this topic to come to an objective conclusion [128]. 

The use of additives in waterjet (WJ) applications has been known since 1972, when Franz [129] outlined the effect of improved coherence due to additives as a method to increase the waterjet penetration depth. A few publications exist in the literature, although a comprehensive study of the different additives effects was not available. Moreover, the application of drag-reducing additives [130,131] to abrasive waterjet (AWJ) technology was not well-developed and understood, especially in the case of injected jets. The aim of the research presented in [128] was to develop an injection system suitable to give a quantitative assessment of the effect of various types of additives on the WJ cutting capability. 

The conclusion of this study indicated how the use of polymeric additives can be recommended in cases of high-precision pure WJ cutting of high-thickness soft materials, such as seals, where a more focused jet can achieve higher productivities and/or higher quality. No advantages were measured in the case of AWJ.

### 5.4. Abrasive Mass Flow Rate and Other Parameters’ Control Performance

Many parameters play an important role on the AWJ cutting quality; some of them are considered process parameters such as pressure, abrasive mass flow rate, feed rate, mesh number, focusing tube and orifice diameter, and others are considered as external factors and are usually neglected in the models such as pressure fluctuations, abrasive mass flow rate fluctuations, cutting head vibrations, workpiece vibrations, abrasive particle size distribution, focusing tube wear etc. The research study reported in [54] dealt with the influence of the AWJ parameters on the cutting performance with the aim of providing a complete overview of the influence of both the process parameters and the external factors explaining how they can contribute to the AWJ cutting performance [2]. This is a prerequisite for understanding which external factors can be put under control, as the abrasive mass flow rate. This consideration led to the development of a suitable optical sensor to acquire the abrasive mass flow rate signal [55] and then a new abrasive mass flow rate closed-loop delivery system [13] (Section 4.1) based on weighting the abrasive hopper on board the cutting head and on a specific actuating valve developed to adjust the shape and dimension of the hole through which the abrasive flow passes before going to the mixing chamber.

Ref. [54] was also important for proposing the idea of modelling and monitoring the parameters that cannot be controlled, as with the water pressure ([16], Section 4.2).

Some studies created the building blocks of microAWJ cutting (Section 6). Some of them are covered by nondisclosure agreements and were useful for two patents on a system for feeding fine abrasives (up to mesh #600) in high-precision or microAWJ [12,13,55,56].

**Figure 21:**
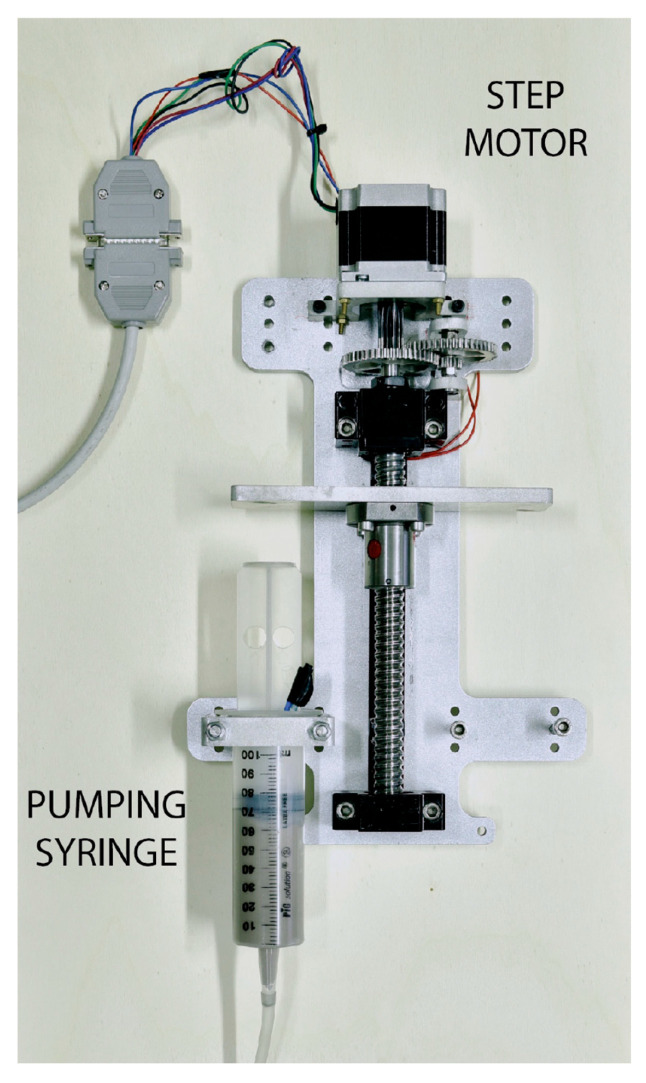
Gel-based abrasive feeding system [132].

In this regard, [14] introduced the possibility to suspend the fine abrasive in a gelatinous water-based fluid. The mass ratio of the dispersed powdered abrasive material to the gelatinous water-based fluid is from 1.0 to 3.5, which means that the slurry is relatively highly loaded. To take this slurry to the cutting head, controlled-stroke pistons (Figure 21) or even peristaltic pumps [15] can be exploited. 

This idea of using a slurry is convenient for some important reasons:

- It prevents abrasive clogging due to wet as the abrasive is already wet;
- It allows for better control of the abrasive mass flow rate by controlling the abrasive concentration in the slurry. With this system, even 5 g/min or less are feasible with an uncertainty of ±1 g/min (see also Section 5.6 for uncertainty evaluation procedures in waterjet-related systems). This capability is due to the fact that it is easy to control the gel flow rate instead of controlling the particle flow rate;
- It allows for the stopping of provided abrasive in a sudden way when it is not needed as the gel flow can be easily stop by blocking the feeding piston or the peristaltic pump. In this way, mixing chamber clogging is prevented;
- Even in the case the loaded gel stays in the mixing chamber after the jet switching off, it can be easily broken by the next jet start. This fact prevents the typical jet clogging due to the presence of stagnant abrasive in the mixing chamber.

### 5.5. High-Pressure Manifolds’ Performance

The design of special components in ultra-high pressure (UHP) applications is a complex matter and it is often accomplished by a trial-and-error approach that can be time and cost consuming with no guarantee of obtaining the desired results. This is true for small series parts or for unique pieces. As known, waterjet components are subject to UHP and must be designed with particular care. The research reported in [133] considers the case of a high-pressure multiorifice manifold installed downstream of the on-off valve (also called needle valve) and the feeding of multiple orifices. The force due to the nominal working pressure, the force oscillating at low frequency (from 0.1 to a few hertz) due to the intensifying pump and the pulsing force produced by the needle valve on-off cycles produce a fatigue stress on the manifold affecting its working life. Special investigation techniques applied in this study have been the finite elements analysis (FEM) [134], useful for pointing out the most stressed areas of the manifold, the failure analysis [135], able to give information on the cracks starting points and on the macro and microgeometrical state of the components close to the critical areas [136], and the dynamic analysis [137], a useful tool for studying the dynamic component behaviour during working operations. Results indicated how particular care has to be paid to the component design (dimensions in particular areas have to be sufficient to produce sustainable stresses, but become useless over certain values), to the stresses produced by the working conditions (peak pressures have to be taken into account) and to the component manufacturing processes and fabrication cycle: deep drilling, standard drilling and reaming, carried out via chip removal or electrical discharge machining (EDM) (or wire electrical discharge machining (WEDM)), must be optimised to not leave large signs that are dangerous for the workpiece fatigue life.

### 5.6. Waterjet Velocity LDV Measurement Performance

The study reported in [138] dealt with the uncertainty evaluation in waterjet velocity measurements carried out by means of a laser Doppler dual-incident beam velocimeter in reference-beam configuration developed at the WJ_Lab. The applied experimental procedure makes it possible to calculate the measurement uncertainty through the determination of its different components [139,140]. Once uncertainty is known, the laser Doppler system is suitable for objective and significant velocity evaluations but also for improvements allowed by the knowledge of the most effective uncertainty sources. Such a subject is typically not considered by the specific waterjet literature but is becoming more and more important due to the evolution of waterjet machining towards high-precision applications (Section 6).

## 6. MicroAWJ

An historically well-known reference paper for waterjet microcutting is [141]. While the evolution of microAWJ at WJ_Lab has been only partially documented in scientific papers as some of the developed concepts have been kept secret to exploit them industrially. 

This section aims to explain this evolution passing through the existing documents and add some other background information that can be useful for a reader interested in understanding how to create value through scientific research in manufacturing.

The topic that opened the way to microAWJ at WJ_Lab was the detailed study of the role of the orifice geometry on the jet stability in time and space.

Section 2 on process modelling explains in detail the logical connection among research studies on orifices, but it is worth setting here the red line towards the microAWJ application.

The orifice studies started from understanding the role of their inner geometry on orifice coefficients [1,20,21,109] to better appreciate the importance of the hydraulic flip phenomenon [121] in creating a stable jet in space (coherence) and time.

The next logical step was to link the orifice hydro-dynamic performance to the cutting performance [4,27]. The interesting fact is that the jet coherence is not always a value in waterjet cutting. When cutting soft materials such as plastic foam with pure waterjets (no abrasive), we proved how a more “disturbed” (less coherent) jet, cuts better as it probably can count on a worse jet surface that acts as the teeth of a broaching tool [29]. 

Around 2010 it was clear how CFD could help in getting a better insight in the jet behaviour and performance [31] so, after studying jet cavitation and hydraulic flip inception [142], ref. [28] investigated the peculiar situation that happens when some droplets remain in the orifice after the jet is switched off, because of capillarity. At the next jet opening, some small droplets can be dragged upwards, pushed up along the orifice tube walls by the incoming air sucked by the jet. In fact, the jet drags the air in contact with its surface out of the orifice, but at the same time it creates a recirculation of air that flows up in contact with the orifice walls (Figure 6).

These upgoing droplets can temporarily clog the sapphire hole creating some instability.

To solve this issue, [29] proposed a new configuration of orifice equipped with a lateral inlet port to create a sort of barrier that protects the capillary part of the orifice and, at the same time, adjusts the jet coherence on purpose.

This enhanced capability of controlling the jet shape (taper) and stability, called “air-assisted pure water jet cutting system” is an important tool for high-precision waterjet cutting [143] (Figure 9).

Some thesis works were devoted to miniaturising the cutting head. No scientific papers published by WJ_Lab deal with this, but it is possible to point out that specific orifices of 50 μm in diameter and specific focusing tubes of 200 μm and less were fabricated by selected providers to make experimental tests.

At the beginning, we started from buying high-end components from the market, such as 50 μm orifices and 200 μm focusing tubes, but then we managed to reduce the focusing tube diameter down to 130 μm.

The reader can check as the ratio between the focusing tube diameter and the orifice diameter is different from the golden rule of waterjet cutting, according to which it should be equal to 3.

**Figure 22:**
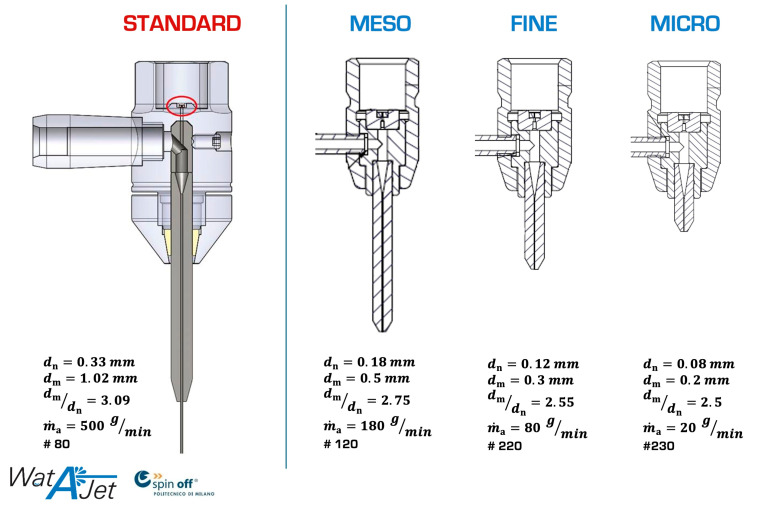
Current configurations of waterjet cutting heads, from standard to microAWJ (courtesy of WatAJet s.r.l., Besnate, Italy). The red circle in the picture points out the position of the orifice.

In fact, current high-precision waterjet plants can count on different cutting heads (Figure 22) depending on the jet size and the accuracy on the part. In the case of microAWJ, the mentioned ratio is smaller (2.5).

**Figure 23:**
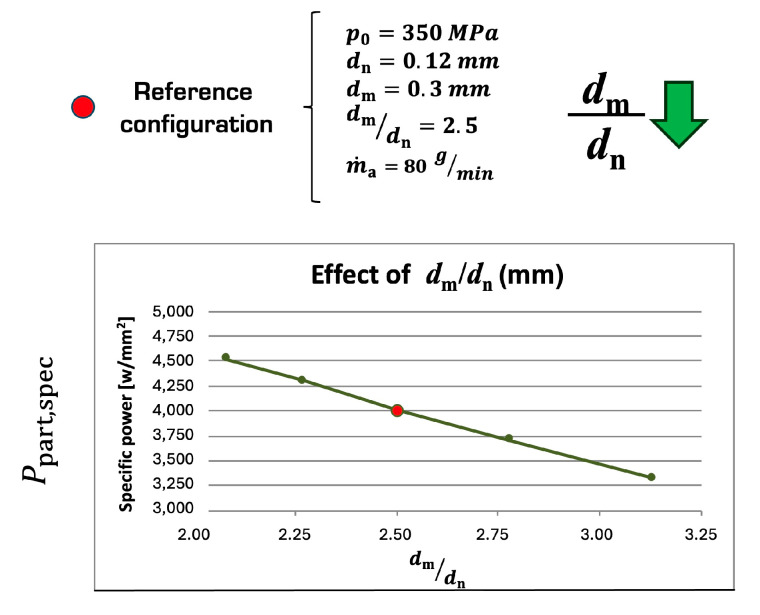
Specific kinetic power of the abrasive particles $P_{\mathrm{part},\mathrm{spec}}$ in function of the focusing tube diameter-orifice diameter ratio. The green arrow indicates how this ratio must be reduced to increase $P_{\mathrm{part},\mathrm{spec}}$.

The explanation for this can be found in the graph of Figure 23 that represents the specific kinetic power [5] of the abrasive particles ($P_{\mathrm{part},\mathrm{spec}}$) in function of the $d_{m}$/$d_{n}$ ratio. $P_{\mathrm{part},\mathrm{spec}}$ increases at smaller values of this ratio as the focuser can better concentrate the kinetic power of abrasive particles on a small area. As flow rates are small in micro scales (down to 5 g/min), this relative reduction in the focusing tube diameter is feasible and effective on the cutting performance. 

The technical knowledge was in place to try to exploit it in an industrial enterprise, so WatAJet s.r.l., Besnate, Italy (www.watajet.com, accessed on 28 February 2024) was founded by the WJ_Lab team (Massimiliano Annoni, Francesco Arleo, Luca Villa and Stefano Volpi).

Some other research studies were carried out in the following years to improve the performance of fine abrasive feeding systems, as described in Section 5 [14,15].

**Figure 24:**
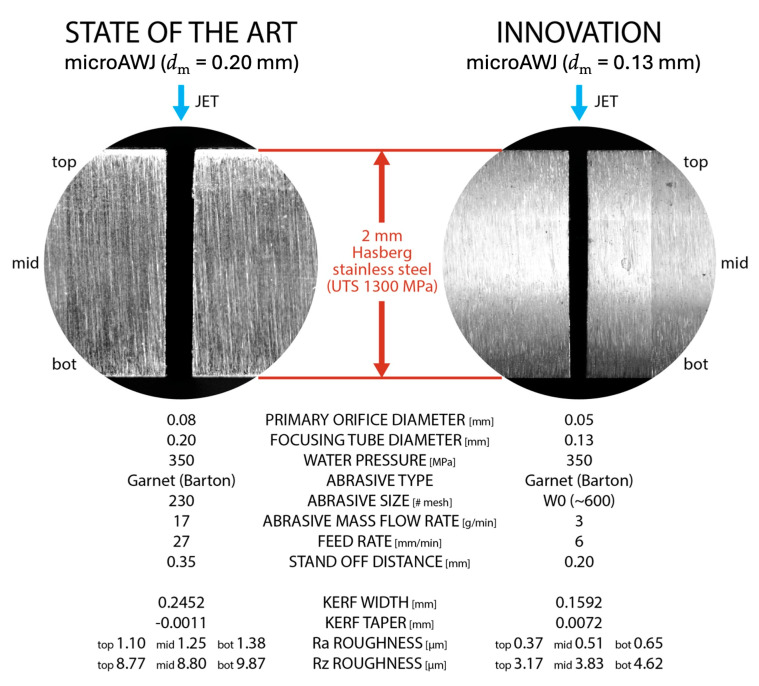
Comparison between the current best performance on the microAWJ market (left) and the finest microAWJ cut carried out at the WJ_Lab [132].

The use of specific abrasive feeders allowed us to come to our finest cut, whose kerf width was less than 160 μm (Figure 24).

**Figure 25:**
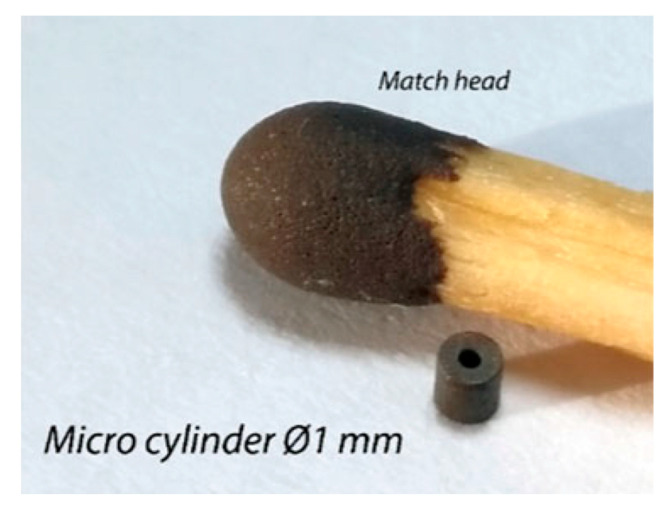
Sintered metal (magnetic steel), thickness = 1 mm, *p* = 340 MPa, Garnet #230, *d*_m_ = 0.2 mm, hole diameter tolerance ± 0.006 (courtesy of WatAJet s.r.l., Besnate, Italy).

A good overview of the microAWJ technology has been published in [7]. This book chapter firstly discusses the main characteristics of the waterjet equipment and the process parameters influencing the final result (Table 5), then a deepened analysis of microAWJ technology is presented along with some case studies to show the technology potential through its actual performance (Figure 25).

Waterjet can now be mentioned among the high-precision and micro manufacturing processes as it can achieve less than 10 μm of dimensional tolerance on the target workpiece. Moreover, it can be placed in high-precision process chains involving other micromanufacturing processes as microWEDM [144,145,146,147].

The capability to manufacture high-precision components with microscale features is enhanced by the combination of different micromanufacturing processes in a single process chain. Ref. [147] explores an effective process chain that combines microabrasive water jet (microAWJ) and micro wire electrical discharge machining (microWEDM) technologies. A spring component was chosen as a leading test case, since fine geometric feature machining and low roughness on the cut walls were required. The advantages deriving from the combination of the two technologies are discussed in terms of machining time, surface roughness and feature accuracy. First, the performances of both processes are assessed via experimentation and discussed. Successively, different process chains are conceived for fabricating two test cases with different size, pointing out some useful indications drawn from this experience.

The experimentation on microAWJ showed that this process is very fast and can obtain valuable surface quality (Ra around 1 μm) and low wall taper. However, the Ra gets slightly worse along the plate thickness. On the contrary, the microWEDM test highlighted that this process reaches finer surface roughness (Ra less than 0.5 μm) without wall taper but with a considerable machining time (hundred times more than microAWJ). For both the test cases, the combination of the two processes fulfils the required surface quality and geometrical accuracy. However, the conceived process chains need a significant machining time, which is affordable only for small parts.

## 7. Applications

As a manufacturing lab, WJ_Lab has always taken the applications of the waterjet process in high regard as advanced use cases are a good way of better understanding the process itself.

This section deals with applications trying to point out their main characteristics and highlighting the role of WJ/AWJ in their solution.

### 7.1. Surface Treatments

One of the characteristics making waterjet a flexible tool is the possibility to adjust the amount of energy released on the target workpiece to pass from a surface treatment as peening [148], to cleaning [149,150], decoating [151,152], milling [153] and then cutting.

The study reported in [154] dealt with WJ decoating, a material removal processes able to strip the surface of the coated components used in the aeronautical field to improve the performance of engines against thermal and mechanical actions.

**Figure 26:**
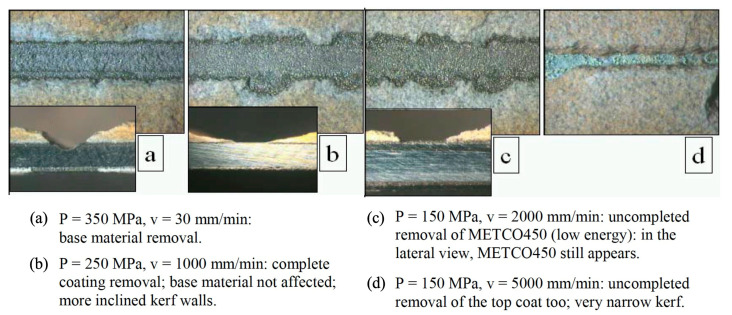
Optical microscope observations (16×) of decoated specimens [154].

The purpose is to decoat complex geometry components without damaging the metallic base material. Chemical treatments are used for this purpose, but they imply a strong environmental impact. Among the mechanical processes, waterjet shows many interesting characteristics able to satisfy the requirements in terms of productivity, flexibility, precision and environmental sustainability. In [154], two different kinds of coating were considered (a thermal barrier and an abradable coating) and an analysis on the waterjet process parameters’ effects on the removal capability was carried out confirming the adequacy of the process (Figure 26).

The study reported in [155] investigated the possibility to perform water jet peening (WJP) [156,157,158,159] by means of a standard waterjet cutting plant. The experimentation was carried out on 39NiCrMo3 specimens with the aim to find out the best working conditions of two different methods: the “in-air WJP” and the “submerged WJP”. Comparisons between the two methods and respect to previous experiments in the reference literature were also presented.

Experiments carried out in this study demonstrate how WJ peening is feasible by employing a standard WJ cutting machine as the achievable residual stress is significant. The two different proposed methods are slightly equivalent in terms of achieved residual stress and optimum SOD, even if the “in-air” process allows higher feed rates and consequently a better productivity. On the other hand, the submerged process is easier to calibrate as the optimum is obtained in a wider range of parameters, while the in-air process needs much more trials to be correctly set. The main disadvantage of both methods is the thickness of the obtained plasticised zone, which is an order of magnitude lower than in case of standard peening processes.

### 7.2. Waterjet Turning

**Figure 27:**
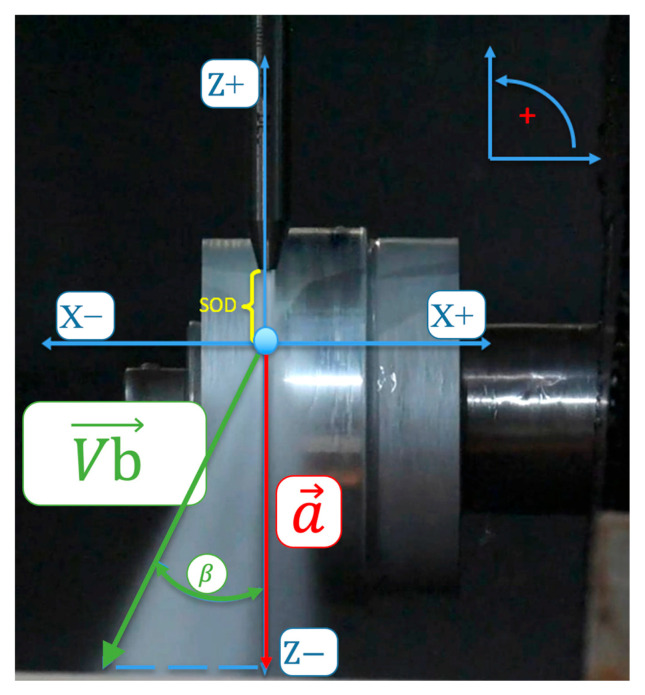
Waterjet turning.

Some WJ_Lab studies have been dedicated to waterjet turning (Figure 27), a niche application of the waterjet process that can be interesting on difficult-to-cut materials [160,161,162,163,164]. Some fundamental studies are helpful to understand the action of abrasive particles on the turned part [165,166].

The effects of the main operational machining parameters on the material removal rate (MRR) in abrasive waterjet turning (AWJT) are presented in [167] using a statistical approach. The five most common machining parameters such as water pressure, abrasive mass flow rate, cutting head traverse speed, workpiece rotational speed and depth of cut (DOC) have been put into a five-level central composite rotatable experimental design (CCRD). 

It has been found that the depth of cut and cutting head traverse speed are the most influential parameters, whereas the rotational speed is insignificant. In addition, this investigation shows that interactions between traverse speed and pressure, abrasive mass flow rate and depth of cut, and pressure and depth of cut are significant on MRR. 

In refs. [168,169], the surface waviness produced by turning aluminium parts with abrasive waterjet was studied. A second order regression model was presented for the waviness. The model validity was confirmed via comparison with experimental data. It was found that abrasive mass flow rate, cutting head traverse speed and DOC are the most influential parameters while water pressure and workpiece rotational speed are less effective.

Finally, the geometrical error in abrasive waterjet turned parts was investigated in [170]. In this paper, a comprehensive study was performed to investigate the influence of machining parameters on the geometrical error (part diameter percent error) in turning AA2011-T4 aluminium alloy round bars. Water pressure, cutting head traverse speed, workpiece rotational speed, abrasive mass flow rate and depth of cut were considered as the main machining parameters in a five-level statistical experimental design. The model predictions were found to be in good agreement with experimental data. Furthermore, among the significant parameters, water pressure, depth of cut and traverse speed are the most influential parameters, with percent contribution of almost 25% each. Abrasive mass flow rate is the least influential parameter with a percent contribution of 4%.

### 7.3. Hybrid Process Chains

Sometimes, waterjet can be employed in process chains involving other processes to get a better overall performance as showed in Section 6 for high-precision applications.

Machining specially shaped geometrical features on hard-to-machine materials is an important task to solve nowadays in manufacturing research and applications. In some cases, two separate technologies can operate on a single feature, exploiting the best performance of both in an optimised process chain. Deep pocket milling is an emblematic case where a combination of AWJ and milling can be used as a close sequential manufacturing strategy. The use of AWJ technology for milling purposes was discussed in [53], considering its advantages and limits compared to conventional milling. This study highlights the potential of coupling these two manufacturing technologies, even into a single hybrid machining centre. A case study on hybrid deep pocket milling on Grade 5 Ti-alloy Ti6Al4V (Ti-64), developed at WJ_Lab, was discussed in [171]. It was demonstrated how this complementary hybrid approach, applied to traditional milling strategies, is more effective in time saving when the pocket depth increases.

**Figure 28:**
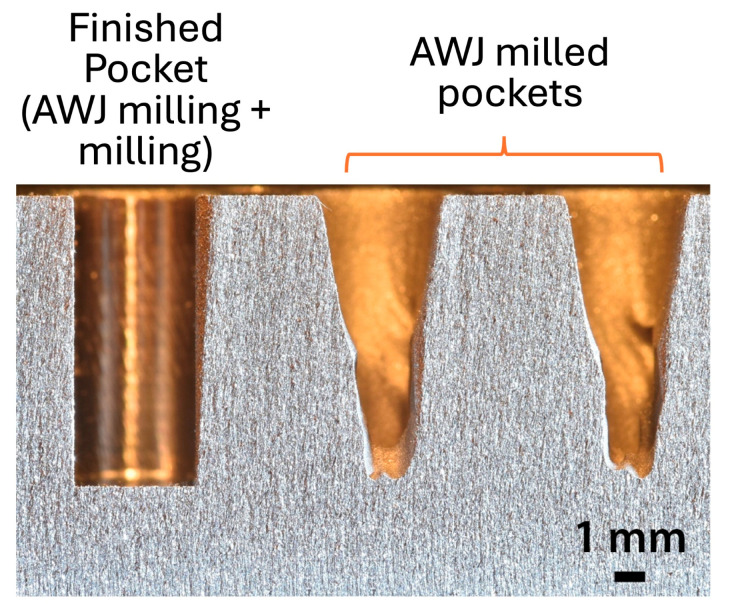
Cross section of an AWJ-milled specimen. The first pocket on the left was finished via milling [172]. Waterjet process parameters: $d_{n}$ = 0.18, $d_{m}=$ 0.50 mm, p = 130 MPa, ${\dot{m}}_{a}$ = 50 g/min, *f* = 46 mm/min, stand off distance = 10 mm, abrasive size = mesh #120.

Also, milling operations on steels for advanced applications entail heavy stress on the tools, causing premature wear and tool breakage at the entrance or in the initial roughing phase. High-precision waterjet cutting was exploited in [172] for the roughing operations thanks to its valuable material removal rate and flexibility (Figure 28) [173,174,175,176,177,178,179]. 

A 4 mm wide and 9.5 mm deep pocket was roughed via AWJ and finished via milling on AISI 415 stainless steel. The main process parameters’ effects were assessed and discussed, as well as the jet path design. The final objective was removing as much material as possible, but still preserving some stock allowance to be removed via milling. The tool wear in the case of plain milling and hybrid machining was inspected, highlighting the convenience of the hybrid solution.

AWJ demonstrates to be capable of removing the most critical portion of the target material at the pocket core region, reducing up to 63% the material for the subsequent standard milling operation.

As already pointed out in Section 6, microAWJ can be effectively coupled with microWEDM in a hybrid process chain [180,181]. The study reported in [182] discusses the performance of microEDM using different flushing media [183,184]. Several tests were performed considering a hardened-steel thin workpieces machined via microEDM with different flushing fluids: deionised water, tap water, deionised water with garnet, tap water with garnet. Garnet is the abrasive material used in microAWJ. Its concentration in microEDM experiments was the same as required in microAWJ machining. A customised system was built on microEDM Sarix SX 200 HP machine (Bozen, Italy) to allow the water-based fluid refill and liquid level monitoring during the experiments. The microEDM trials were carried out considering two machining regimes, roughing and semi-finishing. The different water-based fluids have different electrical conductivity, which leads to different machining performance. Material removal rate (MRR) and tool wear ratio (TWR) were estimated in terms of average and standard deviation. The results show that the presence of garnet does not dramatically affect MRR, since the particles do not play an active role in the erosion process, but it affects surface quality, as proved via the inspection of craters’ morphologies and dimensions estimated with a confocal microscope. MRR generally increased as the conductivity decreased, in particular when semi-finishing regime was used. Furthermore, TWR decreased dramatically with the use of water-based fluids, since a protective recast layer deposited on the tool tip preventing wearing. This analysis shows that microEDM can be successfully performed using the same liquid (water and abrasive) used in microAWJ, paving the way towards the implementation of a hybrid machine based on microAWJ and microEDM technologies.

### 7.4. Cutting of Ceramic Materials

Piezoelectric ceramics are increasingly applied in optical, electronic, mechanical and biomedical applications thanks to their inherent physical properties such as electrical behaviour, electromagnetic response, high temperature strength, hardness and corrosion resistance. Nevertheless, this kind of material is usually very difficult to machine via conventional technologies, while its application becomes more and more demanding in terms of quality and precision. 

A review paper discussed nonconventional and hybrid machining techniques for cutting ceramics, including ultrasonic machining, abrasive jet machining, laser machining and more. It also mentioned comparative studies and recent innovative developments in efficient cutting of ceramics [185].

In such a scenario, fine abrasive waterjets (jet diameter less than 0.5 mm) represent a very appealing and promising technology compared to micromilling, laser or EDM, offering many advantages such as the absence of thermal distortions, high flexibility and versatility, small cutting forces and the increasing capability to cut smaller and smaller features [186,187,188,189]. A research study carried out at Tecnalia R&I in collaboration with WJ_Lab investigated waterjet cutting of thin sheets of a piezoelectric material (PZT, lead titanate zirconate) [190]. A DOE approach was applied to optimise the waterjet cutting parameters and test the capability of the process to be a concrete high-precision technology to machine ceramic materials. Finally, an application of PZT as base material for micro positioning actuators was presented as a case study.

Another study was dedicated to ceramic materials’ cutting in form of ceramic sponge.

**Figure 29:**
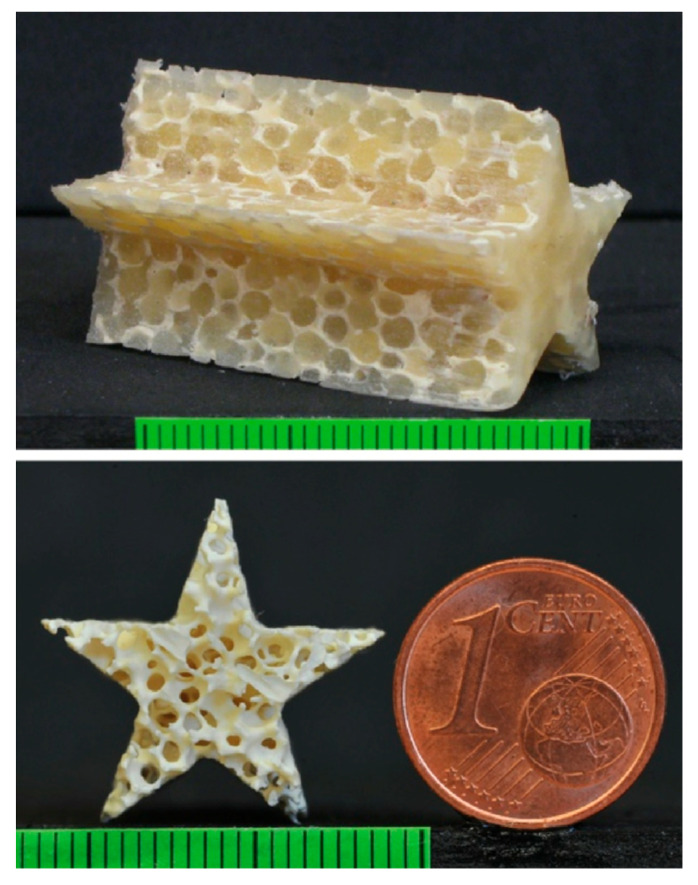
Demonstrative star on 35-mm-thick ceramic sponge before and after filling agent removal [192].

Ceramic sponge machining after firing is a great issue, requiring special tools and procedures because of the material peculiar macrostructure and its intrinsic brittleness [191]. The study reported in [192] approached the problem by exploiting the AWJ process and showing its potential as a flexible tool coming up as an alternative to other ceramic manufacturing processes such as grinding, ultrasonic machining and laser machining [185]. The influence of a temporary pore filling agent, infiltrated in the already sintered sponge, was evaluated and its effect on the jet coherence was investigated through both modelling and experimental approaches. The most suitable process parameters were assessed to reduce the main AWJ defects in these conditions, setting the feed rate at 150 mm/min on a 35 mm thick 30 PPI (pores per inch) ceramic sponge on a conventional cutting equipment. The overall kerf divergence was therefore reduced to less than 1° thanks to the filling procedure and then it was compensated by exploiting a 5-axis AWJ cutting centre. Defects are measured, using both conventional and ad hoc tools (e.g., CMM, grazing light surface inspection and digital image analysis). No thermal or chemical actions are applied by the AWJ cutting process and the negligible forces exerted on the struts preserve their integrity. A case study geometry was machined, fulfilling tight tolerances of 0.1 mm on a Ø 10 mm ceramic sponge cylinder over a 15 mm thickness. A complex-shaped component was cut from a 35-mm-thick sponge (Figure 29).

### 7.5. Nonconventional Waterjet Cutting Applications

In 2003, WJ_Lab carried out a feasibility study on the application of waterjet cutting to rapid prototyping and rapid tooling [193,194]. Waterjet burr formation studies were considered on purpose as burr prevents the correct stacking of layers [195,196]. 

A rapid prototyping technique must satisfy some basic requirements related to the time compression, cost effectiveness and flexibility. The WJ/AWJ technology presents these characteristics especially when building a prototype of big dimensions, no matter of the path complexity or the nature of the target material. In facts, its performance is remarkable in foundry pattern manufacturing, where 5-axis WJ/AWJ systems can reduce the error on sliced objects. 

A laboratory investigation of abrasive waterjet cutting of wheat straws was conducted and reported in refs. [197,198]. The work was aimed at a systematic characterisation of the abrasive waterjet cutting capability of wheat straws, as a potential alternative to cutting discs that are currently adopted in no-till drills and planters for crop residue management [199,200,201,202,203]. A fractional factorial design was applied to investigate the influence of the abrasive waterjet process parameters on the cutting efficiency of wheat straws. Straw coverage thickness, water pressure and orifice diameter were found to be the most significant ones. Experimental results suggest that straw cutting mechanism is mostly related to the hydraulic power of the jet. A multiple logistic regression was performed to model the relationship between the cutting efficiency and the jet power. The logistic model was then applied to estimate the average water and power consumption for wheat straw cutting during a no-tillage seeding operation. An average jet hydraulic power of 6400 W would be sufficiently high to guarantee 90% cutting efficiency in presence of heavy residue distribution. The experimental study shows that a small quantity of abrasive powder (50 g/min) allows to increase the jet cutting capability of wheat straws and reduce the required maximum hydraulic power, compared to pure waterjet cutting. Results are potentially relevant for field validation in no-tillage agriculture.

Characterisation, dismantling and predisposal management of irradiated graphite (i-graphite) have an important role in safe decommissioning of several nuclear facilities which used this material as moderator and reflector [204]. In addition to common radiation protection issues, easily volatising long-lived radionuclides and stored Wigner energy could be released during imprudent retrieval and processing of i-graphite. In this regard, among all cutting technologies [205,206,207], abrasive waterjet (AWJ) can successfully achieve all the thermo-mechanical and radiation protection objectives [82,208]. In [209], factorial experiments were designed and systematically conducted to characterise the AWJ processing parameters and the machining capability. Moreover, the limitation of dust production and secondary waste generation was addressed since they are important aspects for radiation protection and radioactive waste management.

The promising results obtained on nonirradiated nuclear graphite blocks demonstrate the applicability of AWJ as a valid technology for optimising the retrieval, storage and disposal of such radioactive waste. These activities would benefit from the points of view of safety, management and costs.

As a conclusion of this section on waterjet cutting applications, it is worth citing the book “Water jet, a flexible technology” [210]. It is a distillation of the knowledge gained over several decades of research and application of high-pressure waterjet to the cutting, removal and cleaning of a broad range of materials, from bulk rock excavation and nuclear decommissioning to nanotechnology applications in the electronics industry.

The broad base of this technology was built via the studies of the authors included in this book. Their dedication to many combined years of hard work, research, development and application refinement in their respective field, is apparent in this overview of waterjet technology.

## 8. Discussion

The purpose of this section is drawing the main guidelines across the long experience at WJ_Lab with the purpose of highlight the future research topics in waterjet cutting.

The author is convinced that the most important target for researchers dealing with waterjet and considering the industrial improvement of this process as an important objective, is to focus their efforts on a waterjet digital twin according to its definition, i.e., a complete model of machine and process able to exchange data with the real twin with the purpose of:

- Improving their capability of designing new waterjet cutting cycles;
- Improving the final quality on the workpiece in an economical and sustainable way;
- Predicting and preventing damages;
- Programming maintenance.

This trend is already happening for other processes as milling and EDM, while waterjet suffers a certain delay.

This section tries to elaborate on the WJ_Lab experience described in previous sections to demonstrate how it can be reorganised to define a first waterjet digital twin concept.

**Figure 30:**
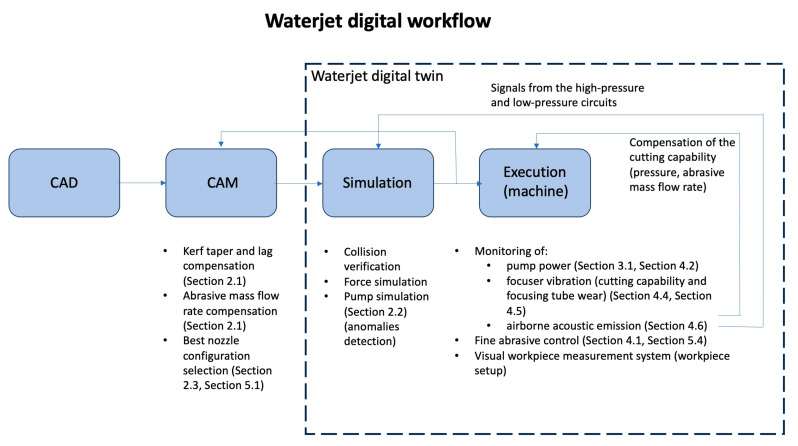
Waterjet digital workflow and digital twin.

Figure 30 helps to guide this discussion. It represents a digital workflow for designing and running a waterjet cutting operation. A part of this workflow can be considered as the waterjet digital twin, as highlighted in the picture.

**Figure 31:**
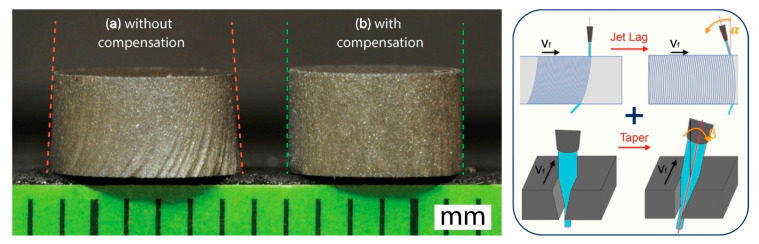
Example of taper compensation obtained at WJ_Lab [132]. Dotted lines indicate the workpiece walls inclination. In the (**b**) case, the workpiece is cylindrical.

The experience on the process modelling applied to WJ and AWJ (Section 2) gave some important insights on the way to the waterjet digital twin:

- CFD is a useful tool for investigating the physical behaviour of fluids and particles inside a cutting head and helps in the design phase of waterjet components. WJ_Lab made use of CFD (Section 2.3, Section 5.1) to better understand waterjet phenomena on the way to microAWJ (Section 6). On the other hand, CFD is not among the building blocks of the waterjet digital twin as, at least for now, it is not fast enough and must be run in an offline mode.
- When dealing with industrial applications, empirical or semiempirical models are more suitable for describing the waterjet-material interaction. In fact, the kerf geometry can be predicted and compensated (Section 2.1) to come to a vertical-wall kerf that can fulfil even tight tolerances, as it happens in microAWJ applications. Compensation can be performed in two ways. The first one is trivial and is based on selecting the proper feed rate to get a null taper. This choice is applied in high-precision waterjet cutting, where productivity is not the primary constraint. Moreover, keeping the feed rate low, as it is needed to get a null taper, is fundamental to keep the surface roughness low, which is always a target in high-precision and in microAWJ. The second way to get a null kerf taper is to incline the cutting head along the feed direction and in the transversal direction [34,105] to compensate for the jet lag and the kerf taper. This solution allows higher feed rates, even if it could lead to high roughness and waviness. Flow Dynamic Waterjet^®^ is a famous patent regarding this concept. WJ_Lab has worked on this kind of compensation with various thesis works (Figure 31), but results are covered by nondisclosure agreements and cannot be published. For sure, both strategies should be embedded in a waterjet CAM system to design manufacturing cycles that can be faster when dimensional tolerance are the main constraint (Figure 30, CAM block).
- A possible refinement of the kerf compensation strategy could be made by adapting the abrasive mass flow rate along the tool path, for example close to the changes of direction (Section 2.1). The CAM system could use this strategy in the manufacturing design phase and a suitable abrasive feeder could carry out a fine abrasive control to this purpose [12,13] (Figure 30, CAM block).
- A waterjet digital twin must include the pump. Section 2.2 describes how the pump was modelled using Modelica. This kind of model could be extremely useful both in the design phase of a new pump, but also as a building block of waterjet digital twin, as it can predict pump signals in different locations along the low-pressure and high-pressure circuits (Figure 30, simulation block). If the model is properly adjusted, it provides the nominal signal that can be used by a monitoring tool to compare with real signals aiming to detect anomalies and stop the machine or program maintenance operations.
- Results of the modelling work on the orifice performance (Section 2.3, Section 5.1) led to the definition of the best orifice geometry for different applications (pure WJ, AWJ, microAWJ). This knowledge should be included in the CAM system to suggest the operator which nozzle configuration to use according to the manufacturing task (Figure 30, CAM block).

Sensorisation (Section 3), monitoring and control (Section 4) are important in digital twins as they are fundamental to acquire information on the real system during operations and then make decisions and act on it to correct drifts, programme maintenance or stop operations. Among the developed sensors and monitoring systems, the most interesting are the noninvasive ones as the three-phase pump power transducer (Section 3.1, Section 4.2, [18]), the instrumented focusing tube (Section 4.4, Section 4.5) and the airborne acoustic emission monitoring system (Section 4.6). The other sensors described in Table 3 are also important, but this discussion focuses only on the above-mentioned sensors and monitoring systems (Figure 30, execution block).

The main considerations on sensorisation, monitoring and control are described here in the following:

- The electric power signal gives the possibility to capture possible unbalances among plungers (Section 4.2), leakages and other pump malfunctions without modifying the pump hydraulic circuit.
- Section 4.4 demonstrates how a AWJ cutting capability index can be extracted from the operational vibrations at the focusing tube. This is an interesting opportunity as it is in general, i.e., nondependent on the workpiece since no sensors need be installed on the target material, and also noninvasive. A monitoring system making use of this kind of instrumented focusing tube could detect drifts in the cutting capability and compensate them by acting on water pressure and/or abrasive mass flow rate, for example by increasing them to keep the cutting capability constant. Section 4.5 explains the use of the same solution to monitor the focusing tube wear. This could give the digital twin the possibility to be aware of the focusing tube status and decide when to stop operations to substitute it.
- An evolution of the focusing tube operational vibration monitoring system is the airborne acoustic emission monitoring system described in Section 4.6. This system is even less invasive.
- Abrasive mass flow rate monitoring and control (Section 4.1, Section 5.4) are possible levers to improve waterjet accuracy and control, so it is advisable to install a closed-loop abrasive feeder on the machine as the ones developed by WJ_Lab [12,13,87,96], also considering previous comments on quality compensation through the abrasive mass flow rate control.
- Laser Doppler velocimetry (Section 3.2) is an important tool for characterising orifice and the overall plant hydraulic performance, but it is not practical in an industrial environment, so it is not included in a possible waterjet digital twin solution.
- Similar considerations can be drawn for force measurements (Section 4.7) that are more suitable for research purposes.

Figure 30 is an important tool for pointing out new functions that should be given to the elements of the waterjet digital workflow:

- CAM systems should not only take care of the tool path and process parameters to cut a certain material in a certain thickness but should suggest the best nozzle configuration depending on the application (target material, pure WJ or AWJ, high precision or standard precision). Moreover, the CAM should plan the cutting head motion, including its inclination to compensate taper and jet lag. A more advanced and futuristic CAM could produce a part program including commands to give to the abrasive feeder depending on the position along the tool path.
- Some CAD/CAM/simulation software capable of simulating waterjet operations already exist on the market. This function is included in the software package offered by waterjet cutting machine builders or in independent software as IGEMS, CGTech Vericut, Autodesk. Current waterjet software can carry out operations as nesting, cycle time calculation, cost evaluation but, regarding the tool path (and machine) simulation, they cannot include the numerical control behaviour, which is important to develop the waterjet digital twin concept. In fact, machine controls are often self-developed or adapted by waterjet machine builders and their working principles are not shared with third party simulation software developers. A complete simulation also including the low-level machine functions is important in digital twins to develop machining cycles that stick to reality and are correct at the first time. Furthermore, simulation should be used in the manufacturing cycle design phase for checking it against collisions. To this purpose, fixtures and parts should be modelled, as it already happens in milling. A future simulation software could even simulate the forces on the workpiece and warn the user in case of criticalities (e.g., workpiece breakage), with the purpose to go back to the CAM and modify the cutting strategy. Another new simulation function could be the possibility to simulate the pump signals. This simulation could be executed offline before the actual machining operation to create the nominal signal patterns to be compared with real ones during the operations. An edge computing equipment could do this job. Further evolutions of the simulation software could also receive other signals from the machine, as the ones indicated in Figure 30 in the execution block.
- The execution on the machine could include the highlighted new functions to close a control circuit on the same machine CNC that should take care of compensating the pressure and abrasive mass flow rate values to keep the cutting capability constant. CNC could receive information on the focusing tube wear status and warn the user. Moreover, it could finely control the abrasive mass flow rate according to the machining cycle designed by the CAM. A bigger control loop could be closed with the simulation block for considering the pump behaviour and improving the pump simulation models. The last point in the execution block of Figure 30 regards new visual workpiece measurement systems that could be installed in the machine to check the position of premachined parts to register the waterjet cutting operations according to existing features. These systems already exist for other processes (e.g., EDM) and are under development at the WJ_Lab for waterjet applications.

To create a suitable communication among the components of the waterjet digital workflow, IIoT should be exploited. This fact requires investments and an open approach of machine builders towards innovation.

## 9. Conclusions

This paper reviews the research work carried out at WJ_Lab (Waterjet Laboratory of the Department of Mechanical Engineering of Politecnico di Milano) over the last 25 years. The research topics have been organised in categories to better highlight the lines according to which WJ_Lab moved in the field of waterjet research and application in this period.

The presented spectra of activities confirms how waterjet is a flexible manufacturing process and how it is still interesting under the physical, engineering and industrial points of view.

Thanks to this review, waterjet research categories seem to converge and form part of a big picture, which is expressed in the discussion section of this paper and is named “Waterjet Digital Twin”.

The term digital twin is already used in many fields of engineering, industry and also other domains, and well represents the ambitious object of WJ_Lab, which is using the developed scientific knowledge on the waterjet process to create a comprehensive model to be used for design, management, execution and maintenance purposes.

Single research categories were studied in the past as they were of scientific and industrial interest per se, but now they can be collected and synthesised under the common umbrella of the digital twin.

It is a powerful concept that can push waterjet research and industrial application even further ahead along the same way that has been already paved by other processes as milling and EDM. Hybrid process chains involving waterjet and other processes will be facilitated by developing a consistent digital twin.

Another topic which comes from this review analysis is microAWJ, or high-precision waterjet cutting. This is a natural evolution of all the manufacturing processes that always try to improve their accuracy and their productivity. Waterjet came after other processes in the high-precision field, but this trend is now clear and industrially important.

These two main concepts (digital twin and micro) will converge towards a unique high-precision, high-productivity and digital waterjet system, able to adapt to different requirements. Artificial intelligence will be then applied to the waterjet industrial field, once enabled by the digital evolution of the process.

The path is still long as it needs a suitable mindset and consequent investments for waterjet players as researchers, the forefront of the evolution, but also machine builders, software developers, service companies and even users. 

For sure, this path is worth following.

## 10. Patents

Patents can be found in references [13,14,15,88,89].

## Figures and Tables

**Table 1:** Governing equations for calculating the waterjet velocity and flow rate (subscripts 0 and 1 indicate Section 0 and Section 1 of the orifice (Figure 4) [1].

$v_{\mathrm{th}}=\sqrt{\frac{2p_{0}}{\rho_{1}}}$	(1)
$v_{\mathrm{th},c}=\sqrt{\frac{2L}{\rho_{1}\left(1-C\right)}\left[{\left(1+\frac{p_{0}}{L}\right)}^{1-C}-1\right]}$	(2)
$\psi =\frac{v_{\mathrm{th},c}}{v_{\mathrm{th}}}$	(3)
$c_{v}=\frac{v_{j}}{v_{\mathrm{th},c}}$	(4)
$v_{j}=c_{v}v_{\mathrm{th},c}=c_{v}\psi \sqrt{\frac{2p_{0}}{\rho_{1}}}$	(5)
$c_{c}=\frac{S_{j}}{S_{0}}$	(6)
$c_{d}=c_{v}c_{c}\psi$	(7)
$Q_{w}=S_{j}v_{j}=c_{c}S_{0}v_{j}=c_{c}S_{0}c_{v}\psi v_{\mathrm{th}}=c_{d}S_{0}v_{\mathrm{th}}=c_{d}\frac{d_{n}^{2}\pi }{4}\sqrt{\frac{2p_{0}}{\rho_{1}}}$	(8)

**Table 2:** Summary of the numerical settings and boundary conditions applied to the model [29].

Model Settings		Pressure–Velocity Coupling	Spatial Discretization		Boundary Conditions	
Multiphase model	VOF	PISO scheme	Pressure	PRESTO!	Water pressure inlet	$p_{\mathrm{up}}$= 200 MPa
VOF scheme	Explicit		Momentum	2nd order	Pressure outlet	$p_{\mathrm{down}}$= 101,325 MPa
Time dependence	Unsteady		Volume fraction	Geo reconstruct	Wall	Shear condition: no slip Water–air contact angle: 76°
Viscous model	Turbulent		Turbulent kinetic energy (κ)	2nd order	Air pressure inlet	1st simulation: $p_{\mathrm{air},1}$= 101,325 MPa 2nd simulation: $p_{\mathrm{air},2}$= 0.3 MPa 3rd simulation: wall
Turbulence model	κ−ϵ realisable					
Body force	Yes		Turbulent dissipation rate (ϵ)	2nd order		
Surface tension	0.0712 N/m					

**Table 3:** Signals acquired on the waterjet plant @WJ_Lab with the employed sensors and tested applications [18].

Signal	Applications	Sensors
Input tensions and currents of the 380 V-50 Hz three-phase induction motor	components characterisation components performance comparison system monitoring and failure detection system control efficiency analysis	Current: LEM; LA−100 P (100 A) Tension: Analog Devices; AD215BY Sensors implemented in a self-produced three-phase power transducer board
Water pressure	pump performance evaluation system monitoring and failure detection efficiency analysis	Intersonde; HP−48 (500 MPa; 0−10 V)
Water volume flow rate	hydraulic power calculations determination of orifices coefficients	Kobold; DPM 1550 G2 L343 (0.05−5 L/min; 4−20 mA)
Water temperature	enthalpy evaluation determination of ambient conditions (important for additives employment)	Thermocouple
Water velocity	components characterisation efficiency analysis	Self-produced LDV system
Oil pressure	intensification ratio calculations	Valcom; 84NC (20 MPa; 0−10 V)
Intensifier piston velocity	pump performance evaluation	Self-produced (0−5 V)
Intensifier piston motion direction	reference for asymmetry detection between intensifier strokes pumping frequency acquisition	Self-produced
Abrasive mass flow rate	acquisition and control	Self-produced closed-loop control system
Volume flow rate of the air entrained into the mixing chamber	abrasive entrainment conditions evaluation abrasive mixing efficiency evaluation	Honeywell; AWM5000 (0−20 slm; 1−5 V)
Pressure of air inside the mixing chamber	abrasive entrainment conditions evaluation abrasive mixing efficiency evaluation	Valcom; 84-R-IC-34 (1000−0 mbar; 0−10 V)

**Table 4:** Governing equations for calculating the specific kinetic power of the abrasive particles [5].

${\dot{m}}_{w}=\rho_{1}Q_{w}$	(9)
$r_{d}=\frac{{\dot{m}}_{a}}{{\dot{m}}_{w}}$	(10)
$v_{\mathrm{abr}}=\frac{v_{j}}{1+r_{d}}$	(11)
$P_{\mathrm{part}}=\frac{1}{2}{\dot{m}}_{a}v_{abr}^{2}$	(12)
$P_{\mathrm{part},\mathrm{spec}}=P_{part}/\frac{d_{m}^{2}\pi }{4}$	(13)

**Table 5:** MicroAWJ process parameters [7] (courtesy of WatAJet s.r.l., Besnate, Italy).

MicroAWJ Process Parameters’ Ranges		Typical Process Parameters (*f*: Feed Rate)
Water pressure (MPa), p	80−380	p= 380 MPa
Water flow rate (L/min), $Q_{w}$	0.3−0.8	$d_{n}=$ 0.10 mm
Orifice diameter (mm), $d_{n}$	0.05−0.10	$d_{m}=$ 0.30 mm
Focusing tube diameter (mm), $d_{m}$	0.20−0.30	Abrasive size = #220 (Garnet)
Focusing tube length (mm), $l_{m}$	20−30 (100·$d_{m}$)	${\dot{m}}_{a}=$ 70 g/min
Abrasive type	Garnet, Olivine, Alumina	Workpiece thickness: 1 mm
Abrasive size (mesh# (µm))	#200 (74) − #350 (44)	Fe alloys: *f* = 200 mm/min
Abrasive mass flow rate (g/min), ${\dot{m}}_{a}$	10−80	Al alloys: *f* = 600 mm/min
Machining area	Up to 800 × 800 mm^2^	Ti alloys: *f* = 260 mm/min
		Carbon fibre: *f* = 800 mm/min

## Data Availability

The raw data supporting the conclusions of this article will be made available by the author on request.

## Nomenclature

**Symbols and acronyms**	
AWJ	Abrasive waterjet
C,L	Constants
CAM	Computer Aided Manufacturing
CFD	Computational Fluid Dynamics
$c_{c}$	Contraction coefficient
$c_{d}$	Discharge coefficient
$c_{v}$	Velocity coefficient
$d_{n}$	Orifice diameter
$d_{m}$	Focusing tube diameter
EDM	Electrical Discharge Machining
*f*	Feed rate
FEM	Finite Elements Method
IIoT	Industrial Internet of Things
LDV	Laser Doppler Velocimetry
microAWJ	Micro Abrasive Waterjet
microEDM	Micro Electrical Discharge Machining
microWEDM	Micro Wire Electrical Discharge Machining
${\dot{m}}_{a}$	Abrasive mass flow rate
${\dot{m}}_{w}$	Water mass flow rate
*Oh*	Ohnesorge number
p	Water pressure
$P_{\mathrm{part}}$	Kinetic power of the abrasive particles (for the sake of brevity jet power)
$P_{\mathrm{part},\mathrm{spec}}$	Specific kinetic power of the abrasive particles (for the sake of brevity jet specific power)
$Q_{w}$	Water volume flow rate
*Re*_L_	Reynolds number of liquid
$r_{d}$	Abrasive loading ratio
$S_{0}$	Nominal cross-sectional area of the orifice
$S_{j}$	Jet cross-sectional area at the vena contracta
$v_{\mathrm{abr}}$	Abrasive waterjet velocity
$v_{j}$	Real jet velocity (or pure waterjet velocity)
$v_{\mathrm{th}}$	Theoretical velocity (Bernoulli)
$v_{\mathrm{th},c}$	Theoretical compressible velocity
WJ	Waterjet
*W*_top_	Kerf width at the top
*W*_bot_	Kerf width at the bottom
ρ	Water density
ψ	Compressibility coefficient
**Subscripts**	
0	Upstream section of the orifice (Figure 4)
1	Downstream section of the orifice (Figure 4)
